# Overexpression of *OsCYP19-4* increases tolerance to cold stress and enhances grain yield in rice (*Oryza sativa*)

**DOI:** 10.1093/jxb/erv421

**Published:** 2015-10-09

**Authors:** Dae Hwa Yoon, Sang Sook Lee, Hyun Ji Park, Jae Il Lyu, Won Seog Chong, Jang Ryol Liu, Beom-Gi Kim, Jun Cheul Ahn, Hye Sun Cho

**Affiliations:** ^1^Sustainable Bioresource Research Center, Korea Research Institute of Bioscience and Biotechnology, Daejeon 305-806, Korea; ^2^Department of Pharmacology, College of Medicine, Seonam University, Namwon 590-170, Korea; ^3^Department of New Biology, Daegu Gyeongbuk Institute of Science & Technology, Daegu 711-873, Korea; ^4^Molecular Breeding Division, National Academy of Agricultural Science, RDA, Jeonju 560-500, Korea

**Keywords:** Apoplast, cold stress tolerance, CYP19-4, immunophilin, increased tillering, *Oryza sativa* L.

## Abstract

AtCYP19-4 (also known as CYP5) was previously identified as interacting *in vitro* with GNOM, a member of a large family of ARF guanine nucleotide exchange factors that is required for proper polar localization of the auxin efflux carrier PIN1. The present study demonstrated that *OsCYP19-4,* a gene encoding a putative homologue of AtCYP19-4, was up-regulated by several stresses and showed over 10-fold up-regulation in response to cold. The study further demonstrated that the promoter of *OsCYP19-4* was activated in response to cold stress. An OsCYP19-4-GFP fusion protein was targeted to the outside of the plasma membrane via the endoplasmic reticulum as determined using brefeldin A, a vesicle trafficking inhibitor. An *in vitro* assay with a synthetic substrate oligomer confirmed that OsCYP19-4 had peptidyl-prolyl *cis-trans* isomerase activity, as was previously reported for AtCYP19-4. Rice plants overexpressing *OsCYP19-4* showed cold-resistance phenotypes with significantly increased tiller and spike numbers, and consequently enhanced grain weight, compared with wild-type plants. Based on these results, the authors suggest that OsCYP19-4 is required for developmental acclimation to environmental stresses, especially cold. Furthermore, the results point to the potential of manipulating *OsCYP19-4* expression to enhance cold tolerance or to increase biomass.

## Highlight

OsCYP19-4 is a novel apoplastic immunophilin that plays a role in developmental acclimation and cold stress, probably via regulation of auxin transport.

## Introduction

Abiotic stresses have adverse effects on plant growth and development, and cold stress in particular significantly constrains the spatial distribution of plants and limits agricultural productivity (Thomashow, 1999). Plants differ in their tolerance to chilling and freezing temperatures. Many important crops of tropical and subtropical origins, such as rice (*Oryza sativa*), maize (*Zea mays*), soybean (*Glycine max*), and cotton (*Gossypium hirsutum*), are sensitive to chilling and are incapable of cold acclimation. Conventional breeding strategies to improve cold stress tolerance in various crop plants have not been successful owing to the complexity of stress tolerance traits, low genetic variation of yield components under stress conditions, and a lack of efficient selection criteria (Sanghera *et al.*, 2011)

Genetic technology has been considered as an alternative tool to develop cold-tolerant plants. Recently, full genome sequencing and related proteomics/metabolomics studies have provided insight into the metabolism involved in cold tolerance (Cui *et al.*, 2005; Li *et al.*, 2011). The mechanisms and genes involved in cold acclimation have been investigated with the aim of producing genetically modified crop plants that are tolerant to cold stress. For example, genes encoding enzymes that modify membrane lipids and transcription factors (Jaglo *et al.*, 2001; Kasuga *et al.*, 1999; Kim *et al.*, 2001; Liu *et al.*, 1998; Sui *et al.*, 2007; Thomashow, 2001) have been tested for their ability to impart cold tolerance to transgenic plants. Nevertheless, the transformation of a single gene appears to have a limited effect on crop freezing tolerance (Thomashow, 2001).

Auxin plays essential roles in various aspects of plant growth and development. On the basis of findings that cold stress affects the polar and lateral transport of auxin (Fukaki *et al.*, 1996; Nadella *et al.*, 2006; Shibasaki *et al.*, 2009; Wyatt *et al.*, 2002) and inhibits the intracellular trafficking of auxin efflux transporters (Shibasaki *et al.*, 2009), it has been suggested that auxin participates in cold stress responses in *Arabidopsis thaliana* (Rahman, 2013). Further investigation is required to elucidate the mechanism by which cold stress is associated with auxin transport.

AtCYP19-4 (also known as CYP5) is an *Arabidopsis* immunophilin (IMM) protein previously identified as interacting with GNOM, which plays an important role in intracellular vesicle trafficking of the auxin efflux carrier pin-formed 1 (PIN1) (Grebe *et al.*, 2000; Saito *et al.*, 1999). The *AtCYP19-4* gene is induced by cold and salt stress but not by heat stress (Saito *et al.*, 1999). Like animal IMMs, plant IMMs comprise two main families, the FK506/rapamycin-binding proteins (FKBPs) and the cyclosporine A (CsA)-binding proteins (CYPs). Despite a lack of structural similarity, these two families share a common peptidyl-prolyl *cis-trans* isomerase (PPIase) domain that catalyses a rate-limiting step in protein folding by *cis-trans* isomerization of proline imidic peptide bonds (Lang *et al.*, 1987; Wang *et al.*, 2010). *Arabidopsis* and rice contain 52 and 56 IMMs, respectively (Ahn *et al.*, 2010; He *et al.*, 2004). The cellular roles and biochemical properties of only some of these IMMs have been characterized (Fu *et al.*, 2007; Gopalan *et al.*, 2004; Lima *et al.*, 2006; Park *et al.*, 2013; Seok *et al.*, 2014; Sirpio *et al.*, 2008). AtCYP19-4 is localized to the endoplasmic reticulum (ER) and displays PPIase and protein refolding activities that are sensitive to the cyclophilin-binding drug CsA, (Anders *et al.*, 2008; Saito *et al.*, 1999). Although AtCYP19-4 showed interaction with the dimerization and CYP binding domain of AtGNOM in yeast two-hybrid and *in vitro* interaction assays, immunofluorescence and immunogold labelling results did not support interaction of the two proteins *in vivo*. Moreover, neither physical nor genetic interaction of AtGNOM with AtCYP19-4 was detected in plants (Anders *et al.*, 2008). Further in-depth study of AtCYP19-4 is therefore needed to elucidate its potential role in auxin transport or PPIase activity.

The aim of this study was to characterize OsCYP19-4, which has previously been reported as a putative ER-localized rice IMM of the CYP family that shows 70% and 68% similarity to AtCYP19-4 and *Chlamydomonas reinhardtii* CYN20-1, respectively (Ahn *et al.*, 2010). *OsCYP19-4* was found to be strongly expressed under environmental stress conditions, especially cold stress. Using green fluorescent protein (GFP) fusion constructs, the authors demonstrated that OsCYP19-4 is localized outside the plasma membrane, most likely in the apoplastic space, and is transported out of the cell via the ER, as determined using the vesicle trafficking inhibitor brefeldin A (BFA). In addition, overexpression of *OsCYP19-4* in rice was shown to confer enhanced tolerance to cold stress and increased tillering capacity. From these results, the authors conclude that *OsCYP19-4* is a promising candidate gene for investigating the complex network involved in cold acclimation and for developing cold-tolerant crops.

## Materials and methods

### Plant materials and growth conditions

Rice (*Oryza sativa* ssp. *japonica* cv. Dong-Jin) seeds were used. The rice seed surfaces were sterilized by treatment with 70% alcohol for 3min, then treated with 50% ROX containing Tween-20 for 40min, and then washed eight times in sterilized water. The seeds were germinated and grown in Yoshida nutrient solution and then transferred to a pot with soil under fluorescent light (12h light/12h dark) at 28 °C and 75% relative humidity in a growth chamber. For the promoter assay, *Arabidopsis thaliana* Columbia-0 ecotype was used; the plants were grown in a growth room under fluorescent light (16h light/8h dark) at 24 °C.

### Gene expression analysis of *OsCYP19-4*

To analyse the expression of *OsCYP19-4* in developing tissues, 1-, 2-, and 6-week-old tissues were collected from rice seedlings or young plants. One-week-old seedlings grown in a growth chamber were harvested and dissected into endosperm, root, and shoot tissues. Two-week-old seedlings were harvested and dissected into endosperm, root, stem, and leaf tissues. Six-week-old plants were harvested and dissected into root, stem, and leaf tissues. To examine the abiotic stress-responsive expression of *OsCYP19-4*, 10-day-old seedlings were treated with 4 °C, 10 °C (cold), 28 °C (mock), 42 °C (heat), desiccation (drought), 10mM hydrogen peroxide (H_2_O_2_), or 100 μM cadmium for 0, 1, 3, 6, 12, and 48 hours. Using RNAiso Plus (TaKaRa, Tokyo, Japan), RNA was extracted from plants grown under normal or stress conditions, and cDNA synthesis was performed as previously described (Ahn *et al.*, 2010) with some modifications. After RNase-free DNase I (RQ1 RNase-Free DNase; Promega, Madison, USA) treatment, 3 µg RNA was used for first-strand cDNA synthesis (RevertAid First-strand cDNA Synthesis Kit; Fermentas, Burlington, Canada). Quantitative real-time PCR (qRT-PCR) was performed on a 7500 Fast reverse transcription PCR instrument (Applied Biosystems, Waltham, MA, USA) using SYBR Premix Ex *Taq* (TaKaRa, Tokyo, Japan) according to the manufacturer’s instructions. The PCRs were performed using the primers listed in Supplementary Table S1 at *JXB* online. All reactions were performed in triplicate. Relative expression levels represent the values relative to that of the corresponding control sample at the indicated time point, after normalization to *OsActin1* transcript levels.

### *OsCYP19-4* promoter analysis

DNA fragments covering the 0.5kb and 2kb upstream of the *OsCYP19-4* coding sequence were amplified from rice genomic DNA by PCR with two pairs of primers (as described in Supplementary Table S1). These PCR fragments were then inserted into binary vector pCAMBIA1381z to create a recombinant transcription unit, *OsCYP19-4 Promoter::β-glucuronidase* (*GUS*). The recombinant *OsCYP19-4 Promoter::GUS* fusion genes were introduced into *Arabidopsis* through *Agrobacterium*-mediated transformation by the floral dipping method (Clough and Bent, 1998). For histochemical GUS assays, T_2_ generation transgenic seedlings were germinated and selected on 1/2 Murashige and Skoog (MS) media containing hygromycin (20mg L^–1^; Duchefa Biochemie, Haarlem, The Netherlands) for 1 week. For cold stress treatment, 1-week-old seedlings were transferred to a cold chamber at 4 °C for 1 d. Histochemical GUS staining was performed as described by Lagarde *et al.* (1996) using the substrate X-Glu (Duchefa Biochemie, Haarlem, The Netherlands).

### Subcellular localization of OsCYP19-4 in plant cells

To express the fluorescent GFP tag fused to the C-terminus of full-length OsCYP19-4 (amino acids 1–208), OsCYP19-4 lacking the signal peptide (amino acids 29–208; OsCYP19-4∆SP), or the OsCYP19-4 signal peptide alone (amino acids 1–30; OsCYP19-4SP), *35S::OsCYP19-4-GFP, 35S::OsCYP19-4∆SP-GFP* and *35S::OsCYP19-4SP-GFP* fusions were constructed in the pCAMBIA1302 binary vector (Supplementary Fig. S3). The gene constructs were transformed into *Agrobacterium tumefaciens* strain GV3101 by the freeze–thaw method (Holsters *et al.*, 1978). The constructs were transiently expressed in *Nicotiana benthamiana* transformed using the agroinfiltration method (Marillonnet *et al.*, 2005). The fluorescent protein expression was examined in epidermal cells 1, 2, and 3 d post-infiltration. For protoplast isolation, the infiltrated lower leaves were scratched with the back of a knife at intervals of 0.5×0.5mm, the midribs were removed, and the leaves were transferred to enzyme solution containing 2% cellulose R-10 (Sigma-Aldrich, St. Louis, USA) and 0.5% macerozyme R-10 (Sigma-Aldrich, St. Louis, USA) dissolved in 0.4M sucrose. After 6h incubation at room temperature, isolated protoplasts were examined by microscopy. Fluorescence imaging was performed using an inverted confocal scanning microscope (LSM510 META; Carl Zeiss, Jena, Germany). The OsCYP19-4SP-GFP construct was co-expressed with the red fluorescent protein (RFP)-labelled ER marker BiP (*35S::SP-RFP-BiP*) (Jin *et al.*, 2001). For membrane staining, tobacco epidermis samples were incubated in 5 μM FM4-64 (Molecular Probe, Waltham, MA, USA) solution for 15min at room temperature. BFA was used to inhibit the secretion of OsCYP19-4 to the apoplast compartment. Transgenic *Arabidopsis* seedlings expressing OsCYP19-4-GFP or GFP protein ectopically were treated with 50 μM BFA for 3h and then co-treated with 5 μM FM4-64 for 15min. Transient expression of OsCYP19-4-GFP in *N. benthamiana* leaves was used to reconfirm the effect of BFA on OsCYP19-4 localization (Supplementary Fig. S4).

### *In vitro* PPIase activity assay

Recombinant His_6x_-OsCYP19-4∆SP (OsCYP19-4 29–208 amino acids, corresponding to the mature protein) was prepared using the pET28a expression vector. Expression was induced by isopropyl β-D-1-thiogalactopyranoside (IPTG) and the 20kDa recombinant protein was purified using the ExiProgen^TM^ protein synthesizer (Bioneer, Daejeon, Korea). The PPIase activity of recombinant OsCYP19-4 mature protein was assayed using the tetrapeptide substrate Suc-AAPF-pNA (*N*-succinyl-Ala-Leu-Pro-Phe-*p*-nitroanilide; Sigma-Aldrich, Ontario, Canada) (Fischer *et al.*, 1984). All reagents were pre-equilibrated to 4 °C. In a 1mL cuvette, 50 or 100nM OsCYP19-4 recombinant protein was mixed with 100 μL α-chymotrypsin (60mg mL^–1^ in 1mM HCl; Sigma-Aldrich, Ontario, Canada) and the volume was adjusted to 975 μL with an assay buffer (40mM HEPES-KOH, pH 8.0, 100mM NaCl, 2mM MgCl_2_, 1mM EDTA). The reaction was initiated by the addition of 25 μL substrate (4mM tetrapeptide in 470mM anhydrous LiCl dissolved in trifluoroethanol). Changes in absorbance due to released *p*-nitroaniline were monitored over a 300 s period, at 390nm, at 10°C, in a Shimadzu UV-2450 spectrophotometer (Shimadzu, Kyoto, Japan) with a thermostatically controlled cuvette holder. CsA, a PPIase inhibitor, was added at a concentration of 1 μM. The experiments were performed three times with different preparations of proteins.

### Generation and molecular analysis of *OsCYP19-4*-overexpressing transgenic plants

For constitutive expression of *OsCYP19-4,* the full-length cDNA sequence of *OsCYP19-4* was inserted into the pCAMBIA1300 vector under the control of the 35S promoter. The construct was transformed into *O. sativa* ssp. *japonica* cv. Dong-Jin by *Agrobacterium*-mediated co-cultivation (Toki *et al.*, 2006). Transgenic seeds were germinated and selected on 1/2 MS medium containing hygromycin (50mg L^–1^) for 1 week and subsequently transferred to pots filled with soil and grown for 4 weeks in a rice growth chamber. Transgenic young plants were planted in a paddy field in mid-May, and the seeds were harvested at the end of October annually for 2 years to obtain T_2_ and T_3_ generations, as described by Kim *et al.* (2014). Genomic DNA isolated from the leaves of transgenic plants (grown in a greenhouse or in a chamber) was analysed by PCR to detect the inserted *35S:OsCYP19-4*, by amplifying the inserted fragments with a forward primer based on the 35S promoter and a reverse primer for *OsCYP19-4* as shown in Supplementary Table S1. Semi-quantitative reverse transcription PCR (RT-PCR) was carried out with gene-specific *OsCYP19-4* primers. DNA and RNA extracted from untransformed wild-type control plants were used as negative controls.

### Cold stress tolerance assays in *OsCYP19-4*-overexpressing plants

Cold stress tolerance experiments were conducted with four independent T_3_ transgenic *OsCYP19-4*-overexpressing lines (OE1–4) and non-transformed wild-type plants, which came from different batches (WT1–4). Rice seeds of the wild-type and *OsCYP19-4*-overexpressing lines were germinated on 1/2 MS medium and 1/2 MS medium containing hygromycin (50mg L^–1^), respectively. The seedlings were transferred to pots and grown in soil for 2 weeks in a growth chamber. For the cold tolerance assay, 2-week-old plants were exposed to 4 °C in a cold room for 4 or 5 d in the dark and then allowed to recover at 28 °C in a growth chamber under 12h light/12h dark conditions. Survival rates were recorded for plants subjected to cold stress for 5 d and allowed to recover for 10 d. Twenty plants from each independent line were used and experiments were replicated three times.

### Electrolyte leakage analysis

Two-week-old seedlings of the wild-type and *OsCYP19-4*-overexpressing (T_3_ generation) lines treated with cold for different numbers of days were subjected to electrolyte leakage (EL) analysis as described by de Los Reyes *et al.* (2013) with some modifications. The leaves were cut into 3cm slices and the relative EL of the leaf slices was measured. The stress-induced EL was calculated using the following formula: % EL stress=EL induced/EL total×100. Relative injury was also expressed as the ratio of EL between stressed and control plants. Three plants from each independent line were used and experiments were replicated three times.

### Yeast two-hybrid and bimolecular fluorescence complementation assay

The DNA regions encoding the mature protein of AtCYP20-1 (At5g58710: amino acids 21–204), AtCYP19-4 (At2g29960: amino acids 24–201), OsCYP20-1 (amino acids 40–220), and OsCYP19-4 (amino acids 29–208) were cloned into plasmid pGBK7. The full-length coding regions for AtRCN1 (At1g25490), AtGNOM (At1g13980; AtGNOM-F), and a fragment encoding the N-terminal residues 1–250 of AtGNOM (AtGNOM-N) were cloned into plasmid pGADT7. The yeast strain AH109 was co-transformed with pGBK7 vector constructs and pGADT7 vector constructs. Transformation with the empty pGADT7 vector was used as a negative control. The clones were screened for interaction on SD/-WLH+1.5mM 3-AT medium. Bimolecular fluorescence complementation (BiFC) assays were performed as previously described by Lee *et al.* (2015). *AtCYP20-1*, *AtCYP19-4*, *OsCYP20-1*, and *OsCYP19-4* full-length cDNAs lacking a termination codon were cloned into pSPYNE-35S, and *AtRCN1* cDNA was cloned into pSPYCE-35S. *A. tumefaciens* GV3101 containing the BiFC constructs and the P19 silencing suppressor plasmid were co-infiltrated into 4-week-old *N. benthamiana* leaves. The leaves were observed for yellow fluorescent protein fluorescence at 24–48h post-infiltration. Confocal laser fluorescence imaging was conducted using a Zeiss R510 confocal laser scanning microscope.

## Results

### Putative CYP19-4 homologues are well conserved and have high similarity to CYP20-1s in monocots

**Fig. 1.**
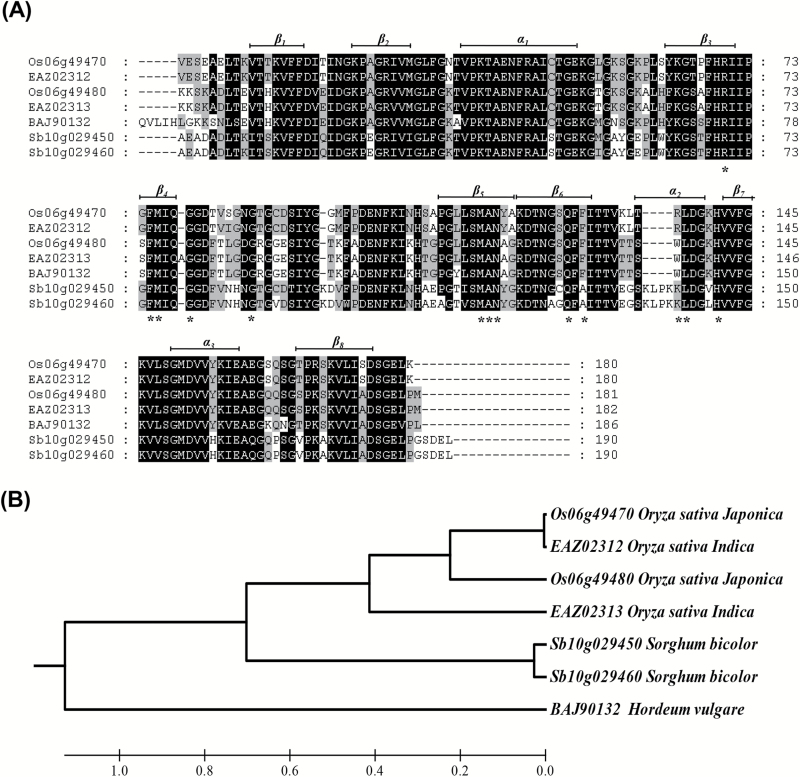
Multiple sequence alignment and phylogenetic relationships among monocot CYP19-4 homologues. (A) Alignment of the mature regions (excluding the N-terminal targeting sequences) of the deduced amino acid sequences of *O. sativa* CYP19-4 and CYP19-4 homologues from other monocot species. The sequences were aligned using ClustalW2 and visualized with GeneDoc version 2.7. Amino acids necessary for CsA binding as determined for human CyPA are marked with asterisks. Secondary structural features (α-helix and β-sheets) are indicated. The backgrounds indicate amino acid similarity: black, 90%; dark grey, 80%; light grey, 40%. (B) Phylogenetic tree of CYP19-4 homologues from monocots. Phylogenetic analysis based on the alignment in (A) was carried out using MEGA5.2.

The *O. sativa* ssp. *japonica* gene *OsCYP19-4* (LOC_06g49470) encodes a protein of 208 amino acids containing a predicted ER signal peptide sequence at the N-terminus and a single PPIase domain; it was classified as a putative homologue of *AtCYP5* (*AtCYP19-4*) in a previous study (Ahn *et al.*, 2010). Putative monocot CYP19-4 homologues were identified by BLASTP searches with rice OsCYP19-4 as the query, and the proteins were aligned using ClustalW2 and visualized by using the GeneDoc program (Nicolas *et al.*, 1997), revealing CYP20-1 cyclophilins to be similar to CYP19-4 proteins among higher plants (Jackson and Soll, 1999). OsCYP19-4 (*Os06g49470*) and OsCYP20-1 (*Os06g49480*) showed high similarity except at the N-terminal signal peptides (75% identity over the mature protein). The N-terminal extension of OsCYP19-4 is predicted to drive vacuole localization, whereas that of OsCYP20-1 is predicted to be a mitochondria-targeting sequence (Ahn *et al.*, 2010). In *Arabidopsis*, CYP20-1 contains a cleavable ER-targeting sequence. Including OsCYP20-1, six putative monocot homologues of OsCYP19-4 were identified. Similar to other CsA-binding domain proteins (Kallen *et al.*, 1991; Ke *et al.*, 1991), the PPIase domain of CYP19-4 homologues contains typical cyclophilin secondary structure features, namely an eight-stranded anti-parallel β-sheet and three α-helix motifs. Furthermore, the 13 amino acids necessary for CsA binding and PPIase activity, as determined for human CyPA (hCyPA) (Zydowsky *et al.*, 1992), were well conserved in all monocot CYP19-4 homologues: 10 of 13 amino acid residues for CsA binding were perfectly conserved (Fig. 1A). Excluding the N-terminal signal peptides, four rice CYP19-4 homologues (including OsCYP20-1; 75% identity) exhibited over 70% identity to OsCYP19-4. The *O. sativa* ssp. *indica* genome was also found to encode OsCYP19-4 (*EAZ02312*: 99% identity to *japonica* OsCYP19-4) and OsCYP20-1 (*EAZ02313*: 74% identity to *japonica* OsCYP19-4), while only one *Hordeum vulgare* CYP19-4 homologue (*BAJ90132*: 72% identity) was identified in the database. *Sorghum bicolor* encoded two CYP19-4 homologues (*Sb10g029450* and *Sb10g029460*).

A phylogenetic tree was constructed on the basis of the mature amino acid sequences of CYP19-4 homologues using the MEGA software program. As shown in Fig. 1B, the phylogenetic analysis revealed that CYP19-4 proteins are highly related to CYP20-1 proteins.

### Expression analysis of OsCYP19-4

**Fig. 2.**
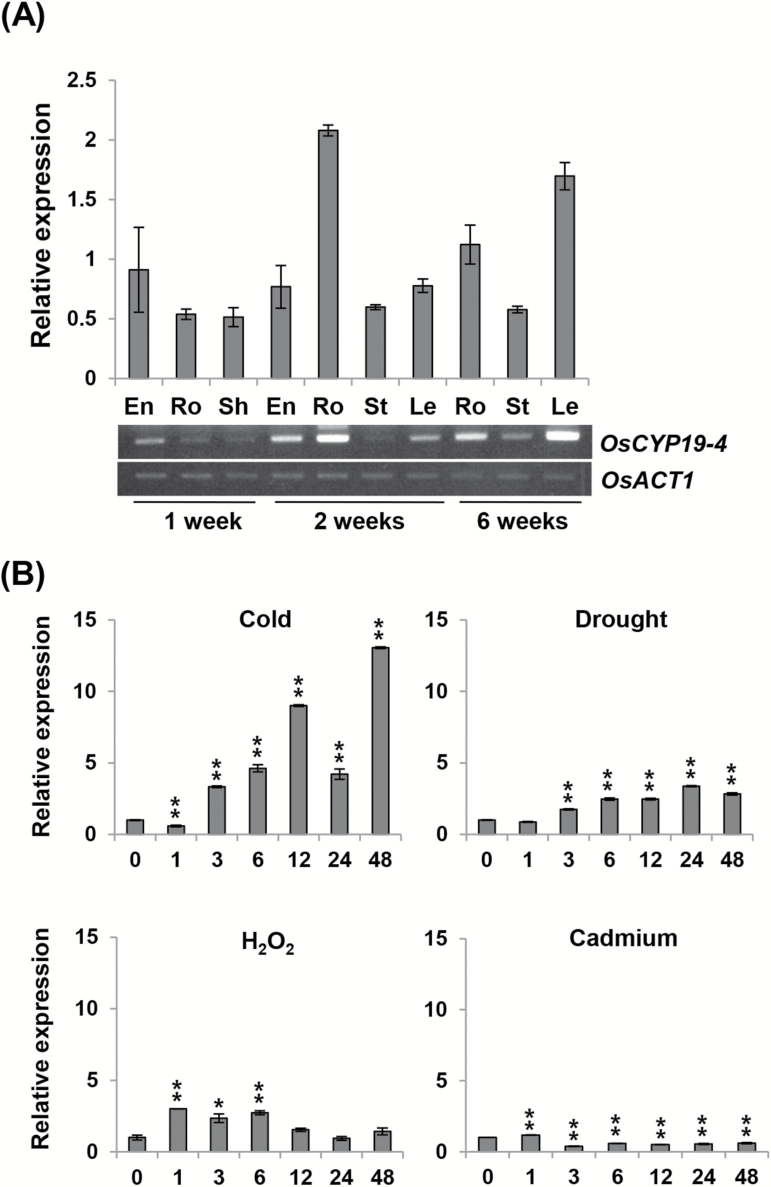
Expression patterns of *OsCYP19-4* in various tissues, developmental stages, and abiotic stress conditions. (A) Expression of *OsCYP19-4* in various tissues and developmental stages, as detected by RT-PCR and qRT-PCR. cDNA templates from endosperm (En), root (Ro), shoot (Sh), stem (St), and leaf (Le) were used for amplification. *OsActin1* was used as a control. (B) qRT-PCR showing the expression of *OsCYP19-4* in 10-day-old rice plants treated with 4 °C (cold), drought, 100mM H_2_O_2_, and 100 μM cadmium for 0, 1, 3, 6, 12, 24, and 48 hours. The relative expressions were normalized to that of *OsActin1*. Error bars denote the SE of three biological replicates. Asterisks indicate significant differences between no treatment (0) and each stress treatment time point (Studentʼs *t* test; **P<0.01).

The expression of OsCYP19-4 in different plant growth stages and tissues was analysed by using RT-PCR and qRT-PCR. In 1-week-old seedlings, *OsCYP19-4* transcript levels were higher in the endosperm than in root and sheath tissues, although expression was not high in any of the tissues (Fig. 2A). Two-week-old plants exhibited sustained expression in the endosperm, with similar levels in the stem and leaves, but much higher expression in the roots. By contrast, in 6-week-old mature plants, *OsCYP19-4* transcript was most abundant in the leaves (Fig. 2A). These results support the notion that OsCYP19-4 functions in various tissues at different stages throughout plant growth and development.

qRT-PCR analysis was performed of *OsCYP19-4* expression under various abiotic stresses, including drought, oxidative stress caused by H_2_O_2_, cadmium, and cold. As shown in Fig. 2B, *OsCYP19-4* transcript increased in response to all treatments except cadmium. Notably, *OsCYP19-4* was most highly induced by cold stress, beginning to increase at 3h and exhibiting a more than 10-fold increase at 48h. Under drought stress conditions, *OsCYP19-4* transcript levels increased starting at 3h, reached a maximum three-fold increase at 24h, and remained up-regulated at 48h of desiccation treatment. H_2_O_2_, which stimulates the generation of reactive oxygen species, rapidly induced a roughly three-fold increase of *OsCYP19-4* expression within 1h after treatment, after which the level decreased over the period from 3h to 48h. However, cadmium, a divalent metal that interferes with the maintenance of auxin homeostasis (Hu *et al.*, 2013), caused reduced *OsCYP19-4* expression from 3 to 48h of treatment. To determine whether the accumulation of *OsCYP19-4* transcripts was affected by temperature, *OsCYP19-4* transcript levels were assessed in plants exposed to temperatures of 4, 10, 28, and 42 °C for various times. The highest level of *OsCYP19-4* transcripts was associated with the treatment at 4 °C; similarly, temperatures of 10 °C (low temperature; maximum five-fold increase) and 42 °C (heat shock; maximum 10-fold increase) were also associated with higher levels of *OsCYP19-4* transcripts compared with the normal condition of 28 °C (Supplementary Fig. S1). *OsCYP19-4* transcript levels were not affected by salinity stress (data not shown).

Together, these results suggest that OsCYP19-4 may function in plant developmental processes in particular tissues at certain stages of growth. In addition, the responsiveness of *OsCYP19-4* expression to various abiotic stresses is consistent with OsCYP19-4 playing a role under stress conditions, particularly cold stress.

### The *OsCYP19-4* promoter is responsive to cold stress in *Arabidopsis*

To examine the cold stress-inducible expression of *OsCYP19-4*, transgenic *Arabidopsis* plants were generated in which the *GUS* reporter gene was driven by the *OsCYP19-4* promoter. *OsCYP19-4* is part of a bidirectional gene pair region, such that the ~2kb region upstream of the *OsCYP19-4* start codon includes ~1.5kb that overlaps with the structure of a gene in the opposite orientation. Predicted *cis*-element regulatory motifs related to cold gene expression regulation were identified in both the full 2kb upstream region of *OsCYP19-4* and the 0.5kb that does not include the bidirectional overlapping region (Supplementary Table S2). Accordingly, both the 0.5kb (Pro-0.5kb) that excludes the overlapping region and the entire 2kb upstream region (Pro-2kb) were cloned and fused with *GUS* (Supplementary Fig. S2A). T_2_ generation transgenic *Arabidopsis* plants were germinated on MS medium for 1 week and then transferred to 4 °C for 1 d for cold stress treatment. GUS activity driven by both the Pro-2kb and Pro-0.5kb *OsCYP19-4* promoters was induced by cold (Supplementary Fig. S2B) compared with untreated controls. This response in a heterologous expression system is consistent with the high induction of endogenous *OsCYP19-4* mRNA under cold stress conditions (Fig. 2B). These results demonstrate that the isolated promoter regions contain *cis*-acting elements involved in up-regulation by low temperature and confirm that *OsCYP19-4* is regulated by cold at the transcriptional level.

### OsCYP19-4 is targeted to the apoplast via the ER in plant cells

**Fig. 3.**
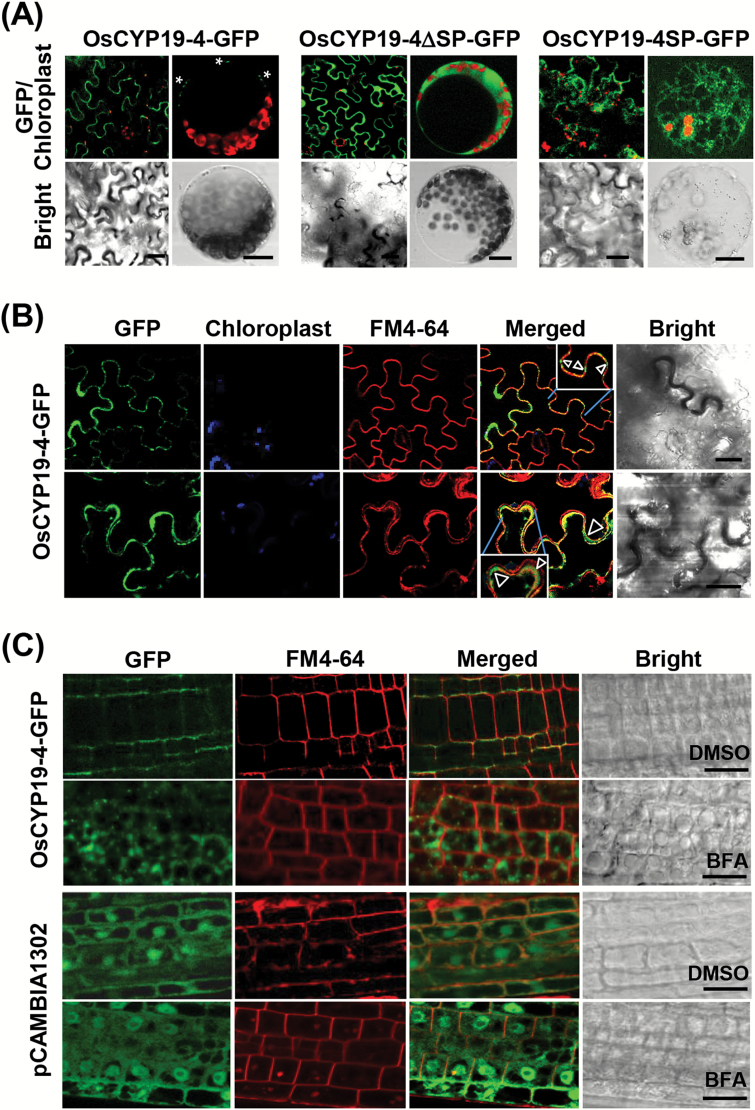
Subcellular localization of OsCYP19-4. (A) Merged images of GFP fluorescence and chloroplast autofluorescence from epidermal cells and protoplasts of *N. benthamiana* infected with *Agrobacterium* GV3101 harbouring OsCYP19-4-GFP, OsCYP19-4ΔSP-GFP, and OsCYP19-4SP-GFP. Asterisks indicate several points of GFP fluorescence near the plasma membrane. (B) Merged confocal laser scanning images of GFP and FM4-64 fluorescence in *N. benthamiana* epidermal cells transiently expressing OsCYP19-4-GFP. The open white arrowheads in the insets indicate OsCYP19-4-GFP protein in the apoplast (green) imaged in the presence of 8.2 μM FM4-64 plasma membrane-staining dye (red). (C) The effect of BFA on the intracellular localization of OsCYP19-4. Transgenic *Arabidopsis* seedlings expressing OsCYP19-4-GFP were treated with DMSO (upper) or the same volume of 50 μM BFA (lower) for 3h and then co-treated with 5 μM FM4-64 for 15min. *Arabidopsis* transgenic seedlings with the empty pCAMBIA1302 vector were used as a control for the BFA compartment assay. Scale bars=20 μm.

The OsCYP19-4 protein was predicted to contain an N-terminal targeting sequence and a transmembrane helix domain between residues 12 and 29 (Supplementary Table S3, Supplementary Fig. S5). An earlier study demonstrated that the AtCYP19-4-GFP fusion protein is detected in the ER around the nucleus, and when the protein is expressed in BY-2 cells, a small amount is secreted into the medium (Saito *et al.*, 1999). To examine the localization of OsCYP19-4 in plant cells, GFP was fused to the C-termini of full-length OsCYP19-4 (OsCYP19-4-GFP), a version of OsCYP19-4 lacking the signal peptide (OsCYP19-4∆SP-GFP), and the predicted signal peptide alone (OsCYP19-4SP-GFP) using a pCAMBIA1302 vector (Supplementary Fig. S3). These constructs were introduced to *Agrobacterium* GV3101 for transient expression in *N. benthamiana* leaves and the resulting fluorescence was observed using confocal laser scanning microscopy. OsCYP19-4-GFP was observed near the boundaries between cells (Fig. 3A). In protoplasts isolated from tobacco expressing OsCYP19-4-GFP, several punctate areas of GFP fluorescence were detected near the plasma membrane (Fig. 3A). By contrast, OsCYP19-4∆SP-GFP signal was localized to the cytoplasm in both epidermal cells and protoplasts (Fig. 3A), similar to the localization of the GFP vector control (Supplementary Fig. S3B). OsCYP19-4SP-GFP transiently expressed in protoplast exhibited a reticular ER pattern (Fig. 3A). In protoplasts, OsCYP19-4SP-GFP signal appeared to be localized to the ER and co-localized with BiP-RFP, an ER marker protein (Supplementary Fig. S3C). These findings suggest that the signal peptide alone may not be sufficient to direct localization of OsCYP19-4 to its final destination.

To characterize the OsCYP19-4 fluorescence detected at the periphery of the plasma membrane of epidermal cells more precisely, and to assess whether OsCYP19-4 was transported into the apoplast, the membrane-selective dye FM4-64 was used. Most of the dye remained on the plasma membrane after 15min incubation at room temperature, and coincided with signal detected in epidermal cells expressing OsCYP19-4-GFP. However, OsCYP19-4-GFP signal was also detected in the apoplast between the plasma membranes of adjacent cells (Fig. 3B). These results suggest that the N-terminal signal peptide of OsCYP19-4 targets the protein to the ER, and that OsCYP19-4 is ultimately transported to the apoplast, probably via the secretory pathway. As BFA blocks export from the ER in plant cells, a BFA compartment assay was conducted using *Arabidopsis* transgenic seedlings expressing OsCYP19-4-GFP and GFP protein. With DMSO (control) treatment, the OsCYP19-4-GFP fluorescence signal was observed in the periphery of the plasma membrane in epidermal cells, as shown in Fig. 3A, B. By contrast, BFA induced redistribution of the distinct OsCYP19-4-GFP fluorescence signals in cytoplasm to structures referred to as BFA compartments (Fig. 3C). The GFP control seedlings showed no difference between the DMSO and BFA treatment conditions. These findings are consistent with those obtained using DMSO or BFA treatment of tobacco leaves transiently expressing OsCYP19-4. In this case, intracellular accumulation of fluorescent OsCYP19-4-GFP was apparent after 3h of BFA treatment. As shown in Supplementary Fig. S4, in the presence of BFA, OsCYP19-4-GFP fluorescence was found in the intracellular space rather than in the apoplast. At higher magnification, the protein could be observed in a network of tubules and planar structures typical of tobacco epidermal ER. Together, these results indicate that OsCYP19-4 is secreted into the apoplast compartment via vesicle trafficking from the ER.

### OsCYP19-4 has PPIase activity in an *in vitro* assay

**Fig. 4.**
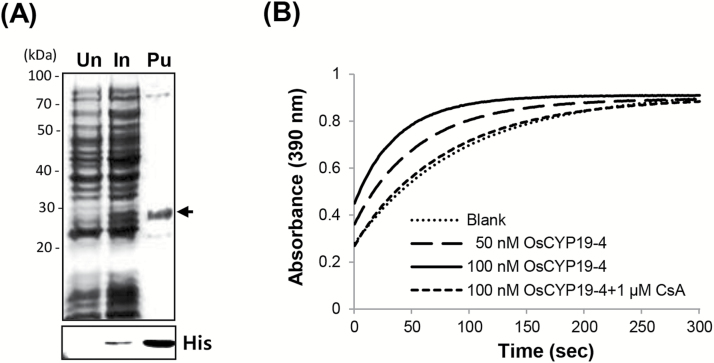
PPIase activity assay using recombinant OsCYP19-4 mature protein *in vitro*. (A) Expression and purification of recombinant mature OsCYP19-4 (OsCYP19-4ΔSP) in *Escherichia coli*. Expression of OsCYP19-4ΔSP in *E. coli* was induced by treatment with IPTG for 4h and the resulting proteins were analysed by 12% SDS-PAGE. Un, un-induced; In, induced by 1mM IPTG for 4h; Pu, purified protein; His, anti-His immunoblot. (B) A protease-coupled assay was used to measure PPIase activity. The prolyl *cis*-*trans* isomerization of the tetrapeptide substrate (Suc-Ala-Phe-Pro-Phe-2,4-difluoroanilide) was reflected by an increase in absorbance at 390nm. The curves represent isomerization of the Suc-AFPF-pNA substrate at 10 °C over the course of 300 s in the absence of OsCYP19-4 (Blank) and in the presence of 50 or 100nM recombinant OsCYP19-4 protein. For the inhibition of OsCYP19-4 PPIase activity, 1 μM CsA was incubated with 100nM OsCYP19-4.

The PPIase domain of OsCYP19-4 includes most of the amino acid residues required for PPIase activity (Fig. 1). To test whether OsCYP19-4 has PPIase activity, a trypsin-coupled assay was conducted, following Kofron *et al.* (1991), using a synthetic tetrapeptide composed of *N*-succinyl-Ala-Phe-Pro-Phe-*p*-nitroanilide (Suc-AFPF-pNA; Sigma-Aldrich, St. Louis, USA) and recombinant His-tagged OsCYP19-4∆SP (corresponding to the mature form of OsCYP19-4; Fig. 4A). This PPIase activity assay is based on the conformational specificity of chymotrypsin, which cleaves the moiety from the synthetic peptide substrate only when the peptide is in the *trans*- conformation (Fischer *et al.*, 1984; Harrison and Stein, 1990; Liu *et al.*, 1990; Siekierka *et al.*, 1989). PPIase activity was assayed at 10 °C in 50mM Tris-HCl buffer (pH 7.5) to suppress the rate of spontaneous thermal isomerization of the peptidyl-prolyl bonds and was measured using absorption at 390nm. A representative spectrophotometric progress curve for the blank condition as a negative control is included in Fig. 4B. The addition of His_6x_-OsCYP19-4∆SP accelerated the *cis-trans* isomerization of the substrate considerably. To test whether the concentration of recombinant protein influenced PPIase activity, a doubled amount (100nM) of recombinant OsCYP19-4 was added to the reaction. A slight increase in PPIase activity was observed with the higher concentration. The addition of CsA blocked the increased PPIase activity of 100nM OsCYP19-4 to the same level as that of the blank (Fig. 4B). Overall, these results show that OsCYP19-4 possesses an active PPIase domain and has PPIase activity *in vitro*.

### Transgenic rice plants overexpressing *OsCYP19-4* show enhanced cold tolerance

To examine the functional significance of OsCYP19-4, transgenic rice plants were generated in which the full-length coding sequence for OsCYP19-4 (amino acids 1–208) was expressed under the control of the CaMV 35S promoter (*OsCYP19-4*-overexpressing lines; Supplementary Fig. S6). Since the expression of *OsCYP19-4* is markedly induced by cold stress (Fig. 2B and Supplementary Fig. S2B), the possible effects of *OsCYP19-4* overexpression on tolerance to cold stress were investigated. Five-day-old *OsCYP19-4*-overexpressing transgenic rice seedlings grown in MS media containing hygromycin were transferred to a growth chamber under 12h light/12h dark cycles at 28 °C in pots with soil and then grown for 10 d. Two-week-old soil-grown plants were moved to 4 °C in the dark for 4 or 5 d for cold stress treatment. Before the treatment, the morphology of the 2-week-old *OsCYP19-4*-overexpressing plants was similar to that of the wild-type plants (Supplementary Fig. S7A). In response to cold treatment, the wild-type plants started to show leaf rolling after 72h and a clear wilting phenotype appeared after 4 or 5 d. Shoots of all four independent *OsCYP19-4*-overexpressing lines (OE1–4) displayed less wilting and rolling in comparison with wild-type plants, and appeared to be relatively healthy under the cold stress conditions (Supplementary Fig. S7B). After treatment at 4 °C for 4 or 5 d, the plants were returned to normal growth conditions. After a 2 d recovery period, wild-type plants that had been treated with cold for 4 d showed a severe stress phenotype: all of the wild-type plants were bent and wilted with rolled leaves and appeared likely to die (Supplementary Fig. S7C). By contrast, the transgenic plants overexpressing *OsCYP19-4* had shoots that were greening, straightening, and healthier than those of the wild-type plants after recovery (Supplementary Fig. S7C). Similar results were observed when plants were allowed to recover for 1 d after a 5 d cold treatment (Supplementary Fig. S7C). These observations support the conclusion that OsCYP19-4 has a specialized function in cold tolerance in rice.

**Fig. 5.**
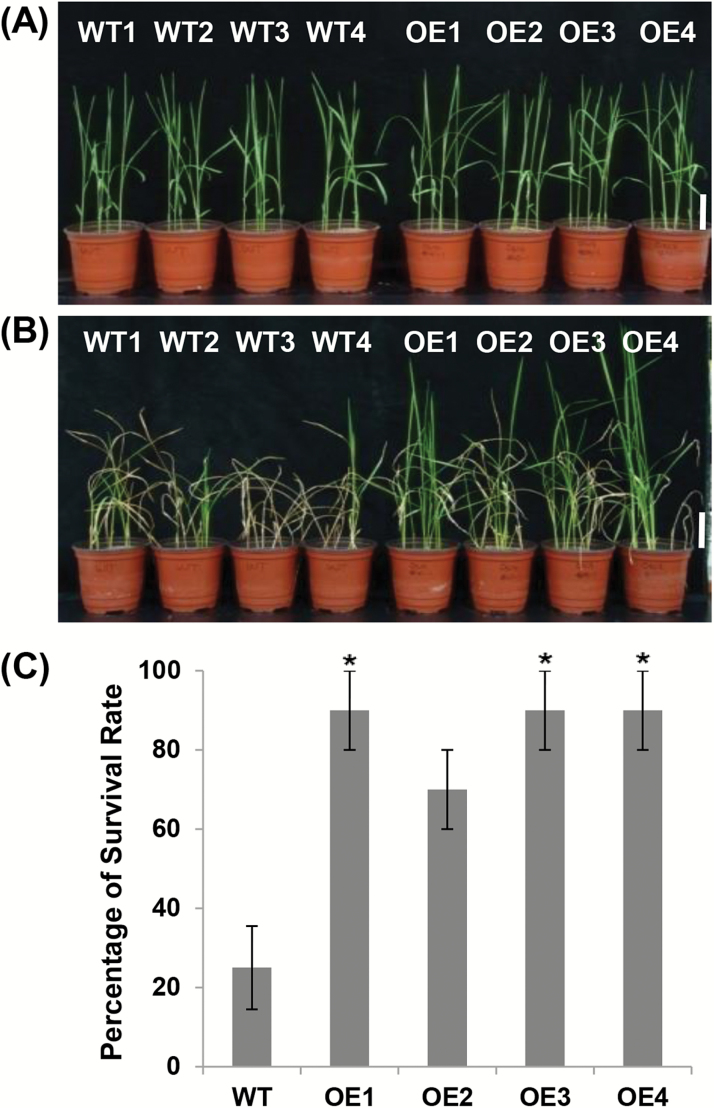
Enhanced tolerance to cold stress in *OsCYP19-4*-overexpressing rice plants. (A) Two-week-old wild-type and transgenic *OsCYP19-4*-overexpressing rice seedlings before exposure to 4 °C treatment under dark conditions for 4 d. (B) Wild-type and *OsCYP19-4*-overexpressing rice seedlings after 4 °C treatment in the dark for 4 d and recovery at 28 °C for 10 d. Scale bars=5cm. (C) Survival rate after cold treatment and recovery. Data are mean±SE of three biological replicates; 20 plants were counted for each of four independent lines. Asterisks indicate statistically significant differences (Student’s *t* test; *P<0.05) between wild-type and transgenic lines.

In addition, to evaluate quantitatively the effect of *OsCYP19-4* overexpression on cold tolerance, the survival rate of the plants in response to cold stress was examined. Two-week-old wild-type and *OsCYP19-4*-overexpressing plants were exposed to a temperature of 4 °C for 4 d under dark conditions. The plants were then transferred to a growth chamber to recover at 28 °C for 10 d under normal light conditions, after which the survival rates were calculated. In line with the previous results, 2-week-old wild-type and *OsCYP19-4*-overexpressing plants showed no difference in phenotype when grown purely under normal conditions (Fig. 5A). By contrast, the cold-treated wild-type and transgenic plants differed greatly in appearance after the 10 d recovery period under normal conditions (Fig. 5B). The survival rate of the wild-type plants was less than 30%, whereas survival rates of the four transgenic lines were much higher: over 70% of the *OsCYP19-4*-overexpressing transgenic plants were green and able to regrow (Fig. 5C). Plants of three of the transgenic lines (OE1, OE3, and OE4) showed a survival rate of nearly 90% and many plants appeared phenotypically similar to the untreated plants (Fig. 5B). This increased survival rate demonstrates that overexpression of *OsCYP19-4* confers cold tolerance to transgenic rice plants. Together with earlier results, these results indicate that OsCYP19-4 plays an important role in cold stress tolerance.

### Low electrolyte leakage of transgenic plants after cold treatment

**Fig. 6.**
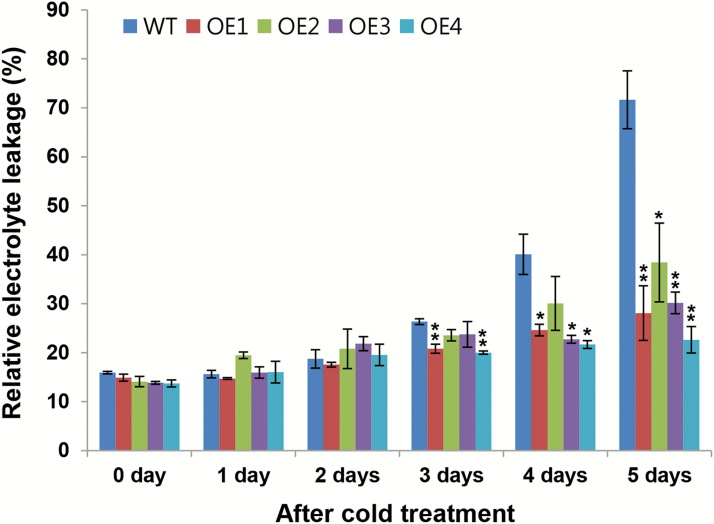
Relative EL between *OsCYP19-4*-overexpressing and wild-type plants under cold stress. Two-week-old rice seedlings were exposed to 4 °C for the indicated number of days and the relative EL of leaf cells was monitored. Data represent mean±SE from three different experiments. Asterisks indicate significant differences between wild-type and transgenic lines (Studentʼs *t* test; *P<0.05, **P<0.01).

Tissue electrical conductivity was measured to assess the degree of membrane injury in plants exposed to cold stress, using leaves of *OsCYP19-4*-overexpressing lines and wild-type plants grown in soil at 4 °C for up to 5 d. The second leaves from the top of 2-week-old *OsCYP19-4*-overexpressing and wild-type plants were used for EL analysis. There was no difference between the wild-type and transgenic plants before the cold stress treatment. For the first 2 d of cold treatment, neither genotype showed any difference in EL compared with EL before the cold stress treatment. On the third day of cold treatment, EL increased in wild-type plants, whereas the *OsCYP19-4*-overexpressing lines showed little change. The physiological state of the wild-type plants thus changed markedly from the third day of cold treatment, and the EL of wild-type plants continued to increase up to day 5 of treatment. This result is consistent with the leaf-rolling and wilting phenotype observed in wild-type plants after cold treatment (Supplementary Fig. S7). After 3 d of cold treatment, the EL of *OsCYP19-4*-overexpressing plants remained lower than that of wild-type plants (ultimately 30% to 50% lower than the wild-type plants by 5 d). The survival rate of the OE2 line was lowest among the transgenic lines, and the EL data were consistent with this physiological result (Fig. 6). These experiments suggest that the enhanced cold tolerance of the *OsCYP19-4*-overexpressing plants is associated with decreased cellular membrane damage and increased membrane stability.

### Enhanced tillering capacity and grain yield in *OsCYP19-4*-overexpressing plants

**Fig. 7.**
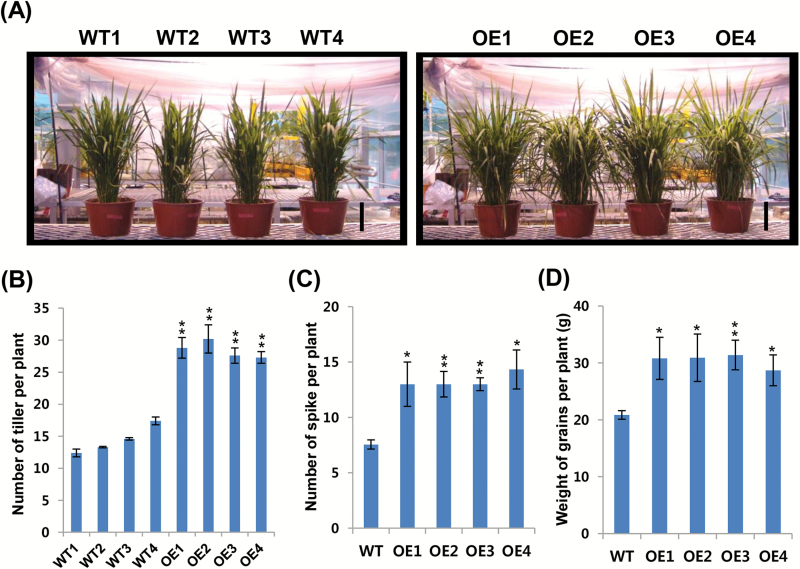
Overexpression of *OsCYP19-4* in transgenic rice plants confers increased tillering capacity and grain yield. (A) Thirteen-week-old wild-type and *OsCYP19-4*-overexpressing plants. Plants were grown in a growth chamber for 4 weeks at the seedling stage and then five plants were transferred to a pot in a greenhouse for 9 weeks (Scale bar=17cm). (B) Number of tillers in 13-week-old wild-type and *OsCYP19-4*-overexpressing plants. Tillers were counted in 20 plants of each line. Data are mean±SE of two replicates. (C) Number of spikes in 32-week-old wild-type and OsCYP19-4 transgenic plants. Data represent mean±SD (*n*=10). (D) Total weight of grains from harvested spikes in 32-week-old wild-type and *OsCYP19-4*-overexpressing transgenic plants. Data represent mean±SD (*n*=10). WT1–4, different wild-type plants; OE1–4, independent *OsCYP19-4*-overexpressing transgenic lines. Asterisks indicate significant differences between wild-type and transgenic lines (Student’s *t* test; *P<0.05, **P<0.01).

Next, the agronomic traits of T_3_ generation *OsCYP19-4*-overexpressing rice plants grown in a greenhouse were monitored. Five-day-old seedlings grown in MS media containing hygromycin were transferred to a growth chamber under 12h light/12h dark cycles at 28 °C in pots with soil and grown for 4 weeks. To examine their phenotypes throughout development, the plants were then transferred to a greenhouse in large plastic pots with soil and grown for 32 weeks. At 13 weeks after germination, the *OsCYP19-4*-overexpressing plants exhibited almost twice as many tillers as the wild-type plants (Fig. 7A, B). The number of tillers per plant typically determines the number of spikes and panicles per plant, and thus tillering is an important trait for grain and/or biomass yield (Li *et al.*, 2010). At harvest stage, the *OsCYP19-4*-overexpressing plants had almost twice as many spikes as wild-type plants (Fig. 7C). Furthermore, the increased tiller and spike numbers of the *OsCYP19-4*-overexpressing plants ultimately led to an increased grain yield (Fig. 7D). These findings indicate that OsCYP19-4 also plays an important role in the control of rice tillering.

### CYP19-4s do not interact with AtGNOM but do interact with AtRCN1, another regulator of auxin polar transport, in yeast two-hybrid and BiFC assays

The physical interaction between CYP19-4s (AtCYP19-4 and OsCYP19-4) and AtGNOM was examined. As rice GNOM has not yet been clearly identified, this experiment used *Arabidopsis* GNOM (AtGNOM) for the yeast two-hybrid assays with CYP19-4s, as well as CYP20-1s (AtCYP20-1 and OsCYP20-1) in light of their high similarity. Yeast cells co-expressing AtGNOM-N (C-terminal deletion construct; amino acid residues 1–250) or AtGNOM-F (full-length construct; amino acid residues 1–1451) with AtCYP19-4, AtCYP20-1, OsCYP19-4, or OsCYP20-1 failed to grow on the selection medium (SD-LTH+3-AT). This result is contrary to previous work in terms of AtCYP19-4 interaction with AtGNOM (Grebe *et al.*, 2000). However, the present study found that AtRCN1 (Michniewicz *et al.*, 2007), another regulator of auxin polar transport and interactor with AtCYP20-1, did interact with CYP20-1s as well as CYP19-4s of both rice and *Arabidopsis*, although there were differences in cell growth on the selection medium (Supplementary Fig. S8A). Next, BiFC was performed to confirm whether AtRCN1 interacts with CYP20-1s as well as CYP19-4s in plant cells. Tobacco cells co-expressing CYP20-1s (AtCYP20-1 and OsCYP20-1) or CYP19-4s (AtCYP19-4 and OsCYP19-4) and AtRCN1 displayed a complementation fluorescence signal (i.e. interaction) properly localized at the stomata and the subsidiary cells. By contrast, cells co-expressing empty vector and AtRCN1 as a negative control did not show any fluorescence (Supplementary Fig. S8B). The expression from all the yeast two-hybrid and BiFC constructs was verified by immunoblot analysis (Supplementary Fig. S9A, B).

## Discussion

A significant number of rice IMM genes are up-regulated in response to environmental stresses. Some of these IMMs have been shown to play a role in acclimation to stress responses in previous studies (Ahn *et al.*, 2010; Kim *et al.*, 2012; Park *et al.*, 2013; Seok *et al.*, 2014). OsCYP19-4 and its putative homologue AtCYP19-4 have previously been reported to be regulated at the transcriptional level by specific stresses (Ahn *et al.*, 2010; Saito *et al.*, 1999). The presence of CYP19-4 proteins in all major monocot plants for which genome sequence data are available suggests that these proteins have important conserved functions (Fig. 1). The present study found that *OsCYP19-4* expression is regulated by various abiotic stresses and is most strongly up-regulated by cold stress (Fig. 2B, Supplementary Fig. S1). *OsCYP19-4* appears to be stably expressed in almost all tissues and stages of development, but is strongly expressed in the root during the growing phase and the leaf during the stationary phase, suggesting that it plays a role spatiotemporally (Fig. 2A). Furthermore, the putative *OsCYP19-4* promoter was responsive to cold stress (Supplementary Fig. S2).

In previous studies, AtCYP19-4-GFP was detected in the ER around the nucleus, Golgi complex, and vesicles, despite AtCYP19-4 lacking any known ER retention signals (Anders *et al.*, 2008; Saito *et al.*, 1999). By contrast, in the present study, it was observed that OsCYP19-4-GFP fusion protein accumulated near the cell periphery or apoplast, as a consequence of ER transport, as determined by a BFA compartment assay (Fig. 3 and Supplementary Fig. S4). The authors are currently carrying out studies to confirm the cellular locations of AtCYP19-4 and AtCYP20-1, another putative ER IMM, as well as OsCYP20-1. Results showed that all the GFP protein fluorescence of AtCYP19-4, AtCYP20-1, and OsCYP20-1 was detected in ER (data not shown); by contrast, OsCYP19-4-GFP fusion protein showed different localization. Further functional analysis both of CYP19-4s and CYP20-1s is required to elucidate their underlying roles in auxin transport or cold-tolerance mechanisms.

The *OsCYP19-4*-overexpressing rice plants displayed enhanced tolerance of cold stress (Supplementary Fig. S7, Fig. 5, 6). In addition, the *OsCYP19-4*-overexpressing plants showed significant enhancement in the number of tillers and spikes, and in grain weight, compared with wild-type plants (Fig. 7). Along with its increased gene expression under cold stress (Fig. 2B), these results strongly suggest that OsCYP19-4 plays a role in crosstalk between developmental acclimation and cold stress. In *Arabidopsis*, AtCYP19-4 interacts with GNOM, which plays a fundamental role in development by regulating endosome–plasma membrane trafficking required for polar localization of the auxin efflux carrier PIN1 (Steinmann *et al.*, 1999), and AtCYP20-1 interacts with the auxin regulator PP2A, AtRCN1 (Michniewicz *et al.*, 2007). The results of the current study suggest that CYP20-1s (AtCYP20-1, OsCYP20-1) and CYP19-4s (AtCYP19-4, OsCYP19-4) can interact with *Arabidopsis* PP2A (AtRCN1), but the interaction with *Arabidopsis* GNOM (AtGNOM) is not clear even with AtCYP19-4 (Supplementary Fig. S8). Therefore, it appears likely that both CYP19-4 and CYP20-1 are linked to regulation of PP2A associated with auxin transport, although it is not known whether the two CYPs exhibit redundancy or whether each has separate functions. The increased tiller number of the *OsCYP19-4*-overexpressing plants (Fig. 7) is similar to that of PIN1 RNAi plants (Xu *et al.*, 2005), suggesting that OsCYP19-4 might affect polar transport of auxin and PIN1 or PIN polar orientation. Given that recombinant OsCYP19-4 showed PPIase activity (Fig. 4), like other known IMMs, the authors hypothesize that OsCYP19-4 also functions through *cis-trans* isomerization of proline residues to regulate the activity of interacting proteins under specific stress conditions such as cold stress, possibly leading to alterations in polar auxin transport.

Overall, this work demonstrates that the overexpression of a single endogenous gene, *OsCYP19-4* confers cold tolerance to rice, as well as increasing grain yield. In addition, the results suggest that *CYP19-4* genes might serve to link developmental responses to stress acclimation.

## Supplementary data

Supplementary data are available at *JXB* online.

Table S1. Primer sequences used in this study.

Table S2. *Cis*-regulatory elements in the *OsCYP19-4* promoter.

Table S3. Predicted subcellular localization of OsCYP19-4 protein.

Figure S1. Levels of *OsCYP19-4* transcripts under various temperature conditions.

Figure S2. Cold-inducible *OsCYP19-4* promoter in *Arabidopsis* transgenic plants.

Figure S3. Localization of OsCYP19-4-GFP in plant cells.

Figure S4. Effects of BFA on the localization of transient expression of OsCYP19-4-GFP.

Figure S5. Predicted transmembrane domain of OsCYP19-4.

Figure S6. Constitutive expression of *OsCYP19-4* in rice.

Figure S7. Response of *OsCYP19-4*-overexpressing rice plants to cold stress.

Figure S8. Interactions of CYP20-1s and CYP19-4s with AtRCN1.

Figure S9. Immunoblot assay of the yeast two-hybrid and BiFC constructs.

Supplementary Data

## Abbreviations:

BFA: brefeldin A
BiFC: bimolecular fluorescence complementation
CsA: cyclosporine A
CYP: cyclosporine A-binding protein
EL: electrolyte leakage
ER: endoplasmic reticulum
FKBP: FK506/rapamycin-binding protein
GFP: green fluorescent protein
GUS: β-glucuronidase
IMM: immunophilin
IPTG: isopropyl β-D-1-thiogalactopyranoside
MS: Murashige and Skoog
PIN1: pin-formed 1
PPIase: peptidyl-prolyl *cis-trans* isomerase
qRT-PCR: quantitative reverse transcription PCR
RFP: red fluorescent protein
RT-PCR: semi-quantitative reverse transcription PCR.
