# Supplementary material for: Overexpression of OsCYP19-4 increases tolerance to cold stress and enhances grain yield in rice (Oryza sativa)
Source: J Exp Bot. 2015 Oct 9;67(1):69–82. doi: 10.1093/jxb/erv421 (PMC4682425; doi:10.1093/jxb/erv421)
Supplement: Supplementary Data [file supp_67_1_69__index.html]

Overexpression of OsCYP19-4 increases tolerance to cold stress and enhances grain yield in rice (Oryza sativa) — Supplementary Data 

# Overexpression of *OsCYP19-4* increases tolerance to cold stress and enhances grain yield in rice (*Oryza sativa*)

## Supplementary Data

Data files

- Supplementary Data - Supplementary Data
