# Supplementary material for: Overexpression of OsCYP19-4 increases tolerance to cold stress and enhances grain yield in rice (Oryza sativa)
Source: J Exp Bot. 2015 Oct 9;67(1):69–82. doi: 10.1093/jxb/erv421 (PMC4682425; doi:10.1093/jxb/erv421)
Supplement: Supplementary Data [file supp_erv421_Supplementary_data.pdf]

Supplementary data - Tables

Overexpression of *OsCYP19-4* increases tolerance to cold stress and enhances grain yield in rice (*Oryza sativa* L.)

Dae Hwa Yoon, Sang Sook Lee, Hyun Ji Park, Jae Il Lyu, Won Seog Chong, Jang Ryol Liu, Beom-Gi Kim, Jun Cheul Ahn, Hye Sun Cho

Table S1. Primer sequences used in is study

Primer Name	Primer sequences<br>(F, forward; R, reverse [5'-3'])	Applications
OsCYP19-4	F : GATCATCCCAGGCTTCATGATCC<br>R : CGCCGCTGTCTGATATGAGGACT	RT-PCR, qRT-PCR
OsACT1	F : CATGCTATCCCTCGTCTCGACCT<br>R : CGCACTTCATGATGGAGTTGTAT	RT-PCR, qRT-PCR
OsHSP101	F : CGGTGGACTTCAGGAACACCGTG<br>R : CGTCGATCTCCTCCTGGATC	qRT-PCR
OsDREB1D	F : CGAAATGGACTACGACACGTACTA<br>R : GATGTCAGCGCCTTCGTC	qRT-PCR
OsCYP19-4	F : GAATTCATGGCGGCGAGGGAGACGTCGCGCCAC<br>R : CTCGAGTCACTTCAGTTCGCCGCTGTCTGATATG	pCAMBIA 1300 (35S)
35S	F : ATGACGCACAATCCCACTAT	g DNA- PCR
	F : AGATCTCATGGCGGCGAGGGAGACGTCGC<br>R : ACTAGTCTTCAGTTCGCCGCTGTCTGATA	pCAMBIA 1302
OsCYP19-4 SP-GFP	R : AATACTAGTTGCCGGCTTGCCATTGATTG	
OsCYP19-4ΔSP-GFP	F : AGATCTCATGGTAGAATCAGAAGCCGAAT	
OsCYP19-4	F : GCATATGATGGCGGCGAGGGAGACGTCG<br>R : CTCGAGCTTCAGTTCGCCGCTGTCTGATA	pET28a
Pro-2.0 kbp	F : GTTGGATCCTTGCATTAGTTTGTCATGGGCAC<br>R : ATAAAGCTTCTCGGATCTCCTCCCGATTCCTC	pCAMBIA1381Z
Pro-0.5 kbp	F : GTTGGATCCCTCCTAATGCATGAGTTGGTTAA<br>R : ATAAAGCTTCTCGGATCTCCTCCCGATTCCTC	pCAMBIA1381Z
AtBiP(SP)-RFP<br>-BiP(C-term)	F : ACCACTAGTTAAATGGCTCGCTCGTTTGGAGC<br>R : AAAGCTAGCCTAGAGCTCATCGTGAGACTC	BiP-RFP construct

Table S2. Cis-regulatory elements in OsCYP19-4 promoter using PLACE

Name of cis-element	Motif	Transcription factor	Stimulus/Tissues	2 kbp	500 bp
-10PEHVPSBD	TATTCT		light	2	1
-300ELEMENT	TGHAAARK		seed	3	1
ACGTATERD1	ACGT	erd1		6	2
ACGTCBOX	GACGTC		seed	2	2
AMYBOX1	TAACARA		amylase,seed	1	
ARR1AT	NGATT	ARR1	cytokinin	15	7
ASF1MOTIFCAMV	TGACG	TGA1,bZIP,CREB	root,leaf,shoot,light,auxin,SA	1	1
AUXREPSIAA4	KGTCCCAT	AuxRE	Auxin,root,meristem	1	
BIHD1OS	TGTCA	rice BELL homeodomain	Binding site of OsBIHD1	11	2
BOXIINTPATPB	ATAGAA	NCII	plastid	1	
BOXLCOREDCPAL	ACCWWCC	MYB		1	1
BS1EGCCR	AGCGGG	Cinnamoyl-CoA reductase	vascular,stem	1	1
CAATBOX1	CAAT	CAAT; legA	seed	27	7
CACTFTPPCA1	YACT	ppcA1	mesohpyll; CACT	39	6
CANBNNAPA	CNAACAC	napA	seed,storage protein	1	
CAREOSREP1	CAACTC	CAREs,GARE	seed	2	1
CARGCW8GAT	CWWWWWW WWG	CArG; AGL15		2	
CATATGGMSAUR	CATATG	SAUR; NDE	auxin	6	
CBFHV	RYCGAC	CBF,CRT/DRE	low temperature	3	3
CCAATBOX1	CCAAT	CCAAT box	HSE (Heat shock element)	9	2
CGACGOSAMY3	CGACG	Amy3D and Amy3E	amylase	3	2
CGCGBOXAT	VCGCGB		calmodulin	2	2
CIACADIANLELHC	CAANNNNAT C		light,leaf,shoot	1	1
CPBCSPOR	TATTAG	POR (NADPH-protochlorophyllide reductase)	cytokinin,chlorophyll,chloroplas t	4	2
CRTDREHVCBF2	GTCGAC	AP2,CRT/DRE; CBF	cold	2	2
CURECORECR	GTAC	CuRE	copper,oxygen,hypoxic	18	2
DOFCOREZM	AAAG	Dof1,Dof2	leaf,shoot	19	3
DPBFCOREDCDC3	ACACNNG	bZIP,ABI5	embryo,ABA,seed	3	
DRECRTCOREAT	RCCGAC	DREB1/CBF	drought,high-light,cold	1	1
E2FBNTRNR	GCGGCAAA	E2Fb,dE2F		1	
E2FCONSENSUS	WTTSSCSS	E2F		1	
EBOXBNNAPA	CANNTG	ABRE,napA	storage protein,seed	30	6
EECCRCAH1	GANTTNC	LCR1	low-CO2	7	
GAREAT	TAACAAR	GARE		1	
GATABOX	GATA	ASF-2	chlorophyll,leaf,shoot	12	3
GCN4OSGLUB1	TGAGTCA	GluB-1	endosperm,seed	1	
GT1CONSENSUS	GRWAAW	GT-1	light,leaf,shoot	10	3

GT1CORE	GGTTAA	rbcS,GT-1,rbcS-3	leaf,shoot	1	1
GT1GMSCAM4	GAAAAA	SCaM-4	GT-1 box	3	1
GTGANTG10	GTGA	pectate lyase	pollen	9	3
HEXAMERATH4	CCGTCG	histone; H4	meristem	1	1
HEXMOTIFTAH3H4	ACGTCA	HBP-1A; HBP-1B; histone H3	meristem	1	1
IBOXCORE	GATAA	rbcS	light,leaf,shoot	2	1
INRNTPSADB	YTCANTYY	psaDb; Inr element	light-responsive transcription	1	
L1BOXATPDF1	TAAATGYA	PDF1,SAM	Shoot apical meristem	1	
LTRECOREATCOR15	CCGAC	LTRE	cold,drought,ABA,leaf,shoot	1	1
MARABOX1	AATAAAYAAA	MAR; SAR	matrix	1	1
MARTBOX	TTWTWTTWTT	MAR; SAR	matrix	1	
MYB1AT	WAACCA	MYB	ABA,leaf,seed	5	2
MYB1LEPR	GTTAGTT	Pti4; ERF; PR; MYB		1	1
MYB2AT	TAACTG	MYB	leaf,shoot	2	2
MYB2CONSENSUSAT	YAACKG	MYB	ABA,leaf,seed	2	2
MYBATRD22	CTAACCA	MYC	Water,ABA,leaf,shoot	1	
MYBCORE	CNGTTR	MYB.Ph3	water,leaf,shoot	3	2
MYBCOREATCYCB1	AACGG	Cyc; M phase; Myb		1	1
MYBGAHV	TAACAAA	Myb,GA,GARC,GARE	seed	1	
MYBPLANT	MACCWAMC	MYB	flower,leaf,shoot	2	1
MYBPZM	CCWACC	MYB	seed	2	2
MYCATERD1	CATGTG	NAC,erd	water-stress	2	1
MYCATRD22	CACATG	MYC	Water,ABA,leaf,shoot	2	1
MYCCONSENSUSAT	CANNTG	MYC,CBF/DREB1,MYB	ABA,leaf,seed,cold	30	6
NAPINMOTIFBN	TACACAT	napin	seed	1	
NODCON2GM	CTCTT	nodulin		5	1
NTBBF1ARROLB	ACTTTA	rolB; Dof	root,shoot,meristem,vascular	1	
OSE2ROOTNODULE	CTCTT	leghemoglobin; Lb29	root,nodule	5	1
POLASIG1	AATAAA	poly A signal; NUE; FUE		6	4
POLASIG3	AATAAT	poly A; polyadenylation		2	2
POLLEN1LELAT52	AGAAA	lat52,MAN	pollen	7	
POLLEN2LELAT52	TCCACCATA	lat52	pollen	1	
PREATPRODH	ACTCAT	bZIP, ProDH		2	1
PROLAMINBOXOSGLUB1	TGCAAAG	GluB-1	seed,endosperm	1	
PYRIMIDINEBOXOSRAMY1A	CCTTTT	Dof,BPBF,GARE	sugar repression,embryo,seed	2	
RAV1AAT	CAACA	RAV1; AP2; VP1; B3	root,leaf,shoot	7	1
RAV1BAT	CACCTG	RAV1; AP2; VP1; B3	root,leaf,shoot	3	
REALPHALGLHCB21	AACCAA	Lhcb21; phytochrome; REbeta		4	2
ROOTMOTIFTAPOX1	ATATT	rolD	root	8	2

RYREPEATBNNAPA	CATGCA	napA	seed	4	4
RYREPEATGMGY2	CATGCAT	glycinin; CATGCAT; Gy2	seed	2	2
RYREPEATLEGUMINBOX	CATGCAY	RY repeat; legumin box	seed	3	3
S1FBOXSORPS1L21	ATGGTA	S1F,RPS1; RPL21	leaf	1	1
SBOXATRBCS	CACCTCCA	rbcS,ABI4	sugar,ABA	1	
SEF3MOTIFGM	AACCCA	SEF3;	seed	2	
SEF4MOTIFGM7S	RTTTTTR	SEF	seed	2	1
SORLIP1AT	GCCAC	phyA	light	2	1
SORLIP2AT	GGGCC	phyA	light	6	2
SURECOREATSULTR11	GAGAC	sulfate transporter	ARF	2	
SV40COREENHAN	GTGGWWHG		SV40; core	1	
TAAAGSTKST1	TAAAG	KST1,Dof	guard cell	5	1
TATABOX2	TATAAAT		legA	1	
TATABOX3	TATTAAT		sporamin	1	1
TATABOX5	TTATTT		glutamine; synthetase	3	1
TBOXATGAPB	ACTTTG	GAPB	light-activated	2	
TGACGTVMAMY	TGACGT		cotyledon,seed germination,seed	1	1
WBBOXPCWRKY1	TTTGACY	WRKY		1	1
WBOXATNPR1	TTGAC	NPR1; WRKY; WRKY18		3	1
WBOXHVISO1	TGACT	WRKY,SUSIBA2	sugar; SURE	5	1
WBOXNTCHN48	CTGACY	WRKY	elicitor	2	
WBOXNTERF3	TGACY	ERF3	wounding	6	1
WRKY71OS	TGAC	WRKY,MYB	TGAC	18	4
XYLAT	ACAAAGAA			1	

Table S3. Predicted subcellular localization of OsCYP19-4

Program	Prediction	Cleavage site
SignalP (4.1)	Signal peptide	Between positions 28 and 29
TargetP (1.1)	Secretory pathway	Signal peptide (1-28 aa)
Predotar	ER	
GolgiP	Golgi body (Transmembrane domain model)	
Golgi Predictor	Golgi Type II membrane protein	
WoLF PSORT	Vacuole	
PSORT (6.4)	Vacuole (0,809) / Outside (0.771)	

SignalP (4.1) sever (http://www.cbs.dtu.dk/services/SignalP/), TargetP (1.1) server (http://www.cbs.dtu.dk/services/TargetP/), GolgiP server (http://csbl1.bmb.uga.edu/GolgiP/), Golgi predictor sever (http://ccb.imb.uq.edu.au/golgi/golgi_predictor.shtml), Predotar (http://urgi.versailles.inra.fr/predotar/predotar.html), WoLFPSORT Prediction (http://wolfpsort.seq.cbrc.jp/), PSORT (6.4) Prediction (http://psort.hgc.jp/form.html).

Supplementary data - Figures

Overexpression of *OsCYP19-4* increases tolerance to cold stress and enhances grain yield in rice (*Oryza sativa* L.)

Dae Hwa Yoon, Sang Sook Lee, Hyun Ji Park, Jae Il Lyu, Won Seog Chong, Jang Ryol Liu, Beom-Gi Kim, Jun Cheul Ahn, Hye Sun Cho

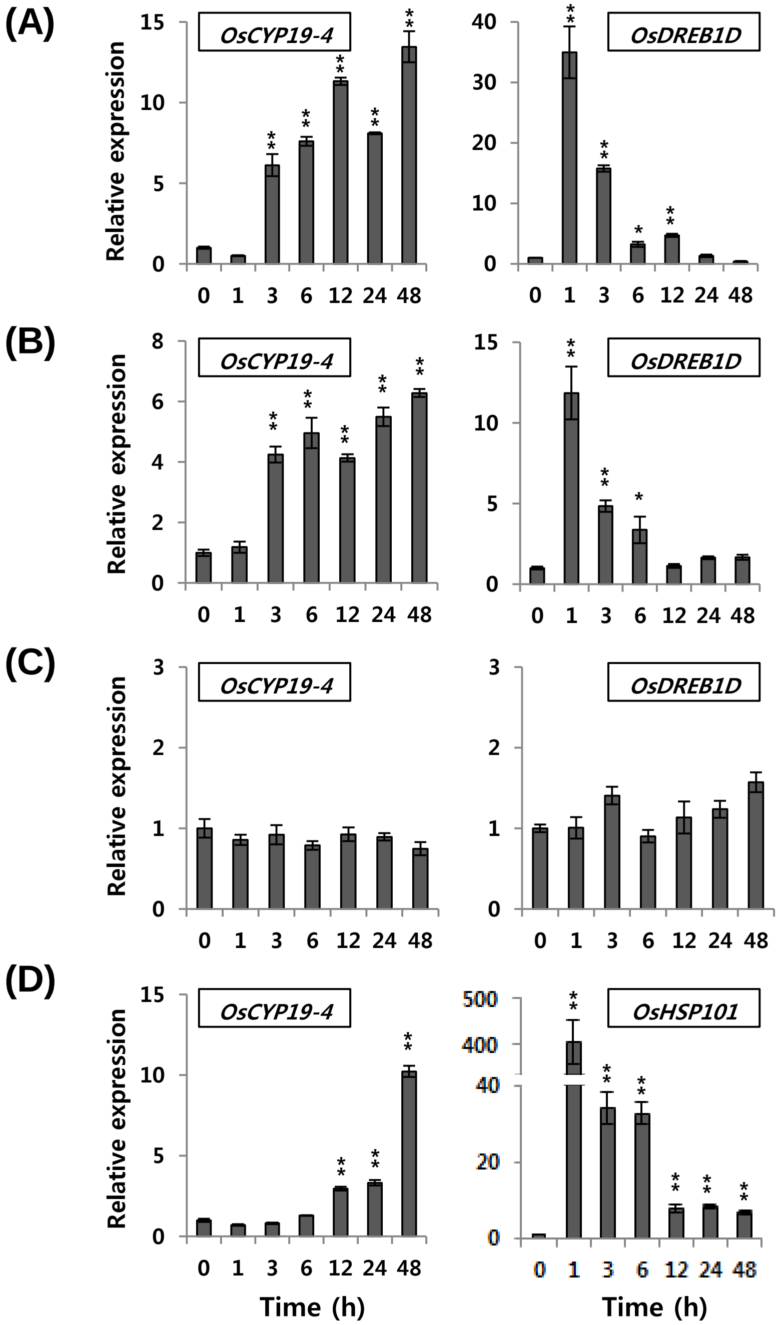

Figure S1. Expression of *OsCYP19-4* transcripts under various temperature conditions. Ten-day-old rice seedlings were treated with various temperature stresses. (A) 4℃, (B) 10℃, (C) 28℃, (D) 42℃. Total RNA was extracted from the seedlings and analyzed by quantitative real-time PCR. The relative expressions were normalized using a *OsACT1* expression. Asterisks indicate statistically significant differences between no treatment (0) and each stress treatment time points (Student's *t*-test; *p < 0.05, **p < 0.01). *OsDREB1D* and *OsHSP101* were used as marker genes, cold and heat stress, respectively.

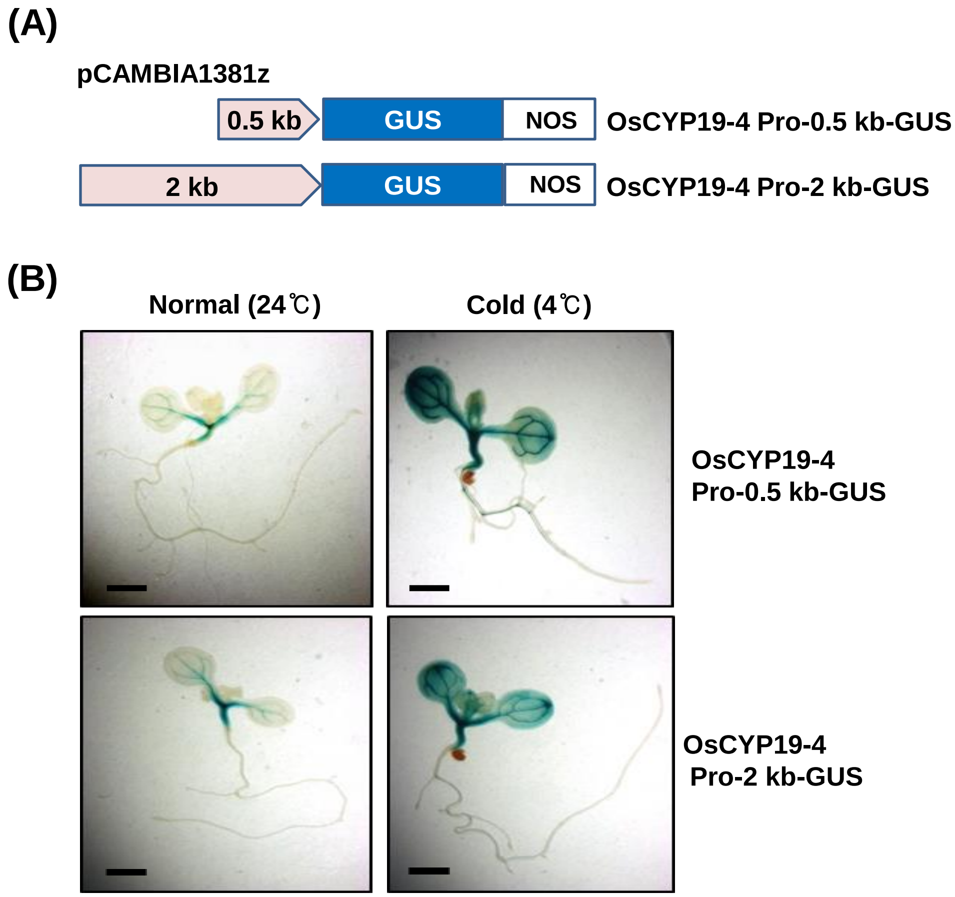

Figure S2. Cold inducible *OsCYP19-4* promoter in Arabidopsis transgenic plants. (A) Structure of the binary vector constructs used for GUS activity analysis of the Pro-2 kb and Pro-0.5 kb *OsCYP19-4* promoter-*GUS* fusions. (B) Histochemical GUS staining of 1-week-old Arabidopsis transgenic seedlings harboring the indicated *OsCYP19-4* promoter-*GUS* constructs. Seedlings were maintained under normal growth conditions or treated at 4°C for 1 d. Twenty seedlings were selected on MS media containing hygromycin of the independent three lines for the constructs and represented the typical results of one of them under normal and cold stress conditions. Scale bars = 2 mm.

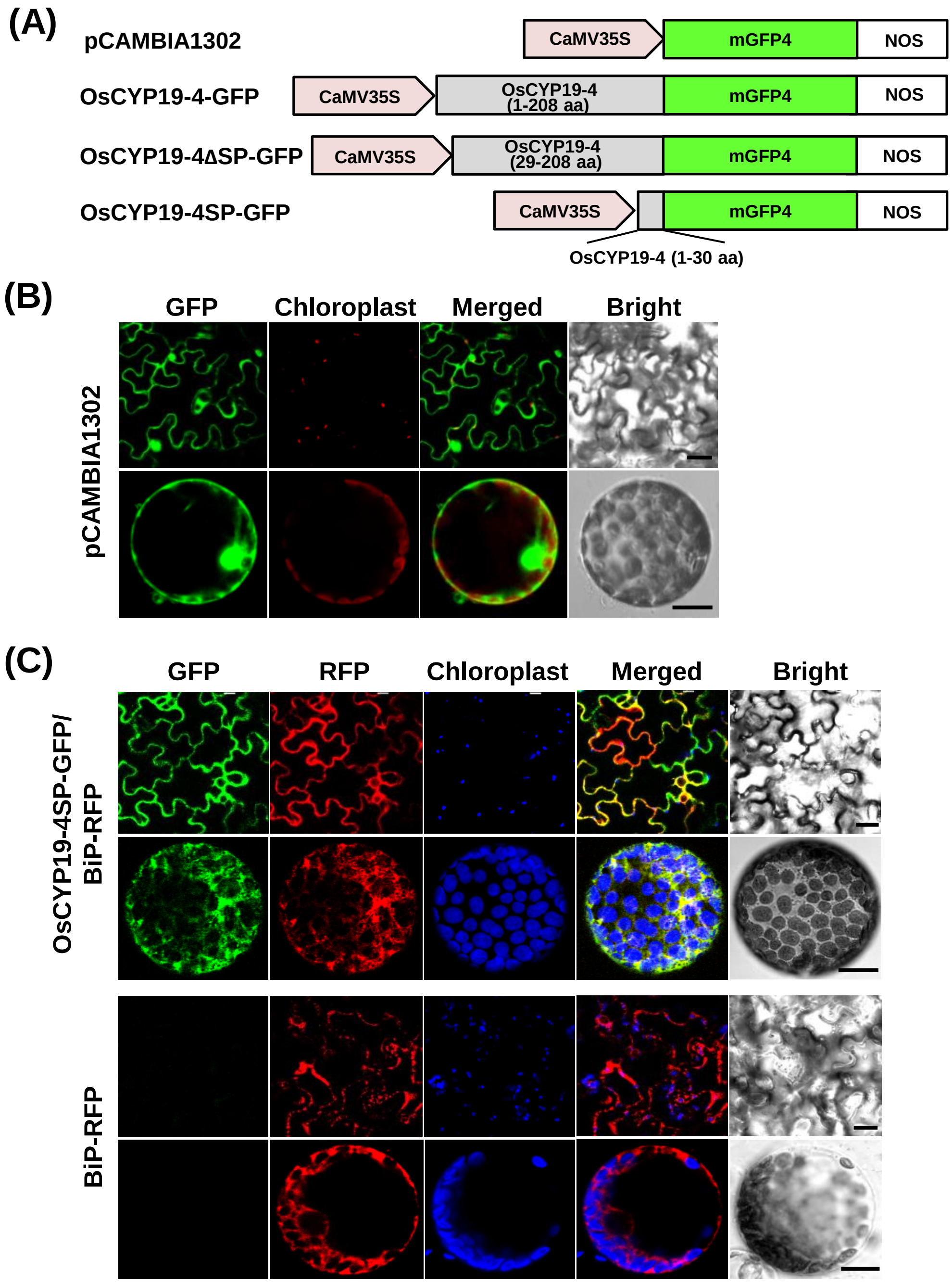

Figure S3. Localization of OsCYP19-4-GFP in plant cell. (A) Structures of the pCAMBIA1302 binary constitutive expression vector harboring the full OsCYP19-4 ORF (OsCYP19-4-GFP) or a version deleted in the coding sequence for the putative signal peptide (OsCYP19-4ΔSP-GFP), and a version only transit peptide of OsCYP19-4 (OsCYP19-4SP-GFP) for transient expression in *N. benthamiana* leaves. (B) Confocal microscopic images of pCAMBIA1302 GFP protein, as a control, from epidermal cells (upper) and protoplast (lower) of *N. benthamiana* leaves. (C) Co-localization of OsCYP19-4SP-GFP and BiP-RFP, as an ER marker, in transient expression *in N. benthamiana* from epidermal cells (upper) and protoplast (lower). BiP-RFP was expressed as ER a marker. Scale bars = 20 μm.

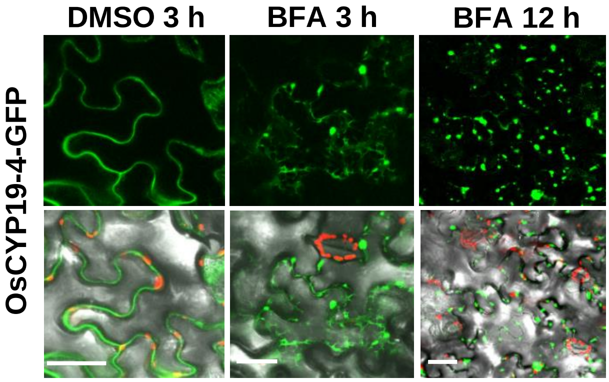

Figure S4. The effects of BFA on the localization of transiently expressed OsCYP19-4-GFP. OsCYP19-4-GFP was expressed in the epidermal cells of *N. benthamiana* and 48 h later, they were treated with 50 ug mL^{-1} BFA or the same volume of DMSO for 3 h using infiltration method. Scale bars = 20 μm.

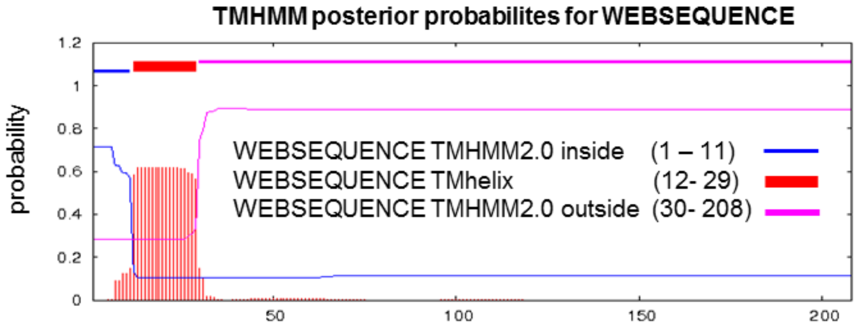

Figure S5. Transmembrane prediction of OsCYP19-4 protein using the program TMHMM Server v. 2.0 (http://www.cbs.dtu.dk/services/TMHMM-2.0/).

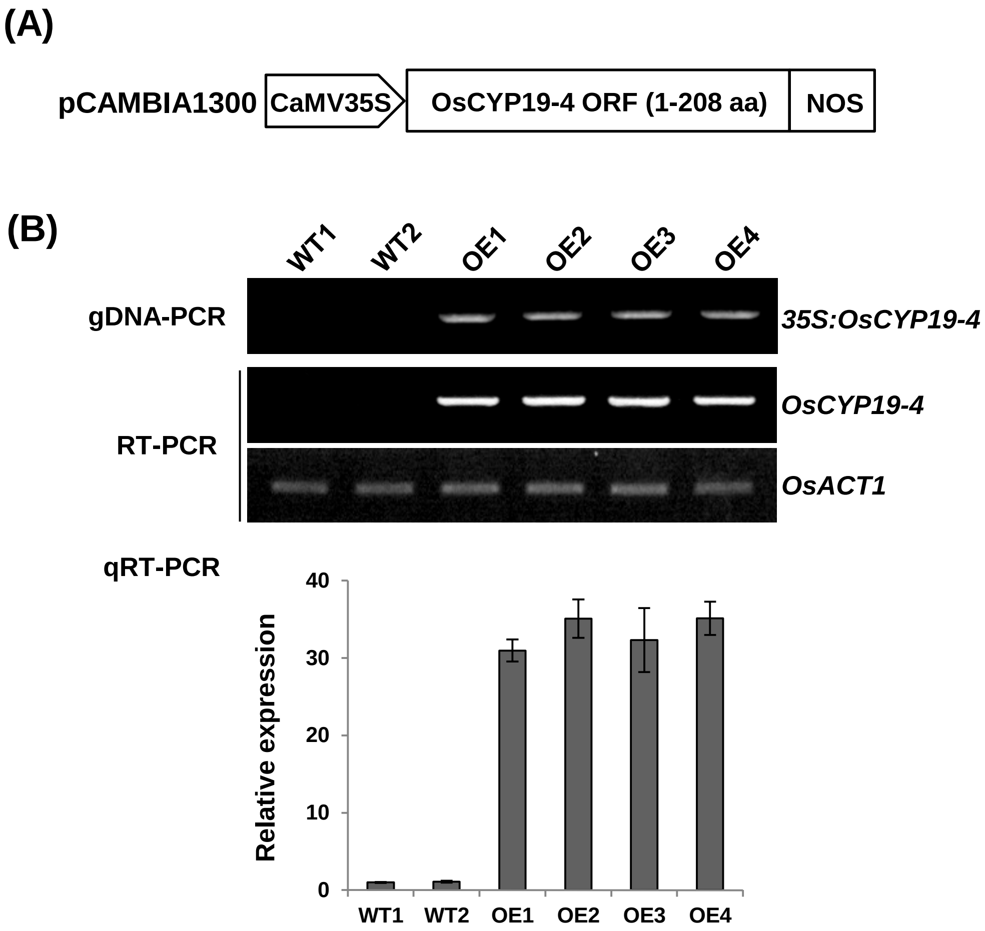

Figure S6. Constitutive expression of *OsCYP19-4* in rice. (A) Structure of the pCAMBIA1300 binary vector containing *OsCYP19-4* under the control of the 35S promoter. (B) Overexpression of *OsCYP19-4* in rice transgenic plants. gDNA-PCR was performed with genomic DNA from wild-type and OsCYP19-4 overexpressing transgenic plants using CaMV 35S promoter and an OsCYP19-4 gene-specific primer set. RT-PCR and qRT-PCR were performed with wild-type control and *OsCYP19-4* overexpressing transgenic plants. *OsACT1* was used as a control for mRNA normalization. WT1 and WT2, wild-type Dong-Jin cultivar plants. OE1-OE4, *OsCYP19-4* overexpressing independent transgenic rice lines.

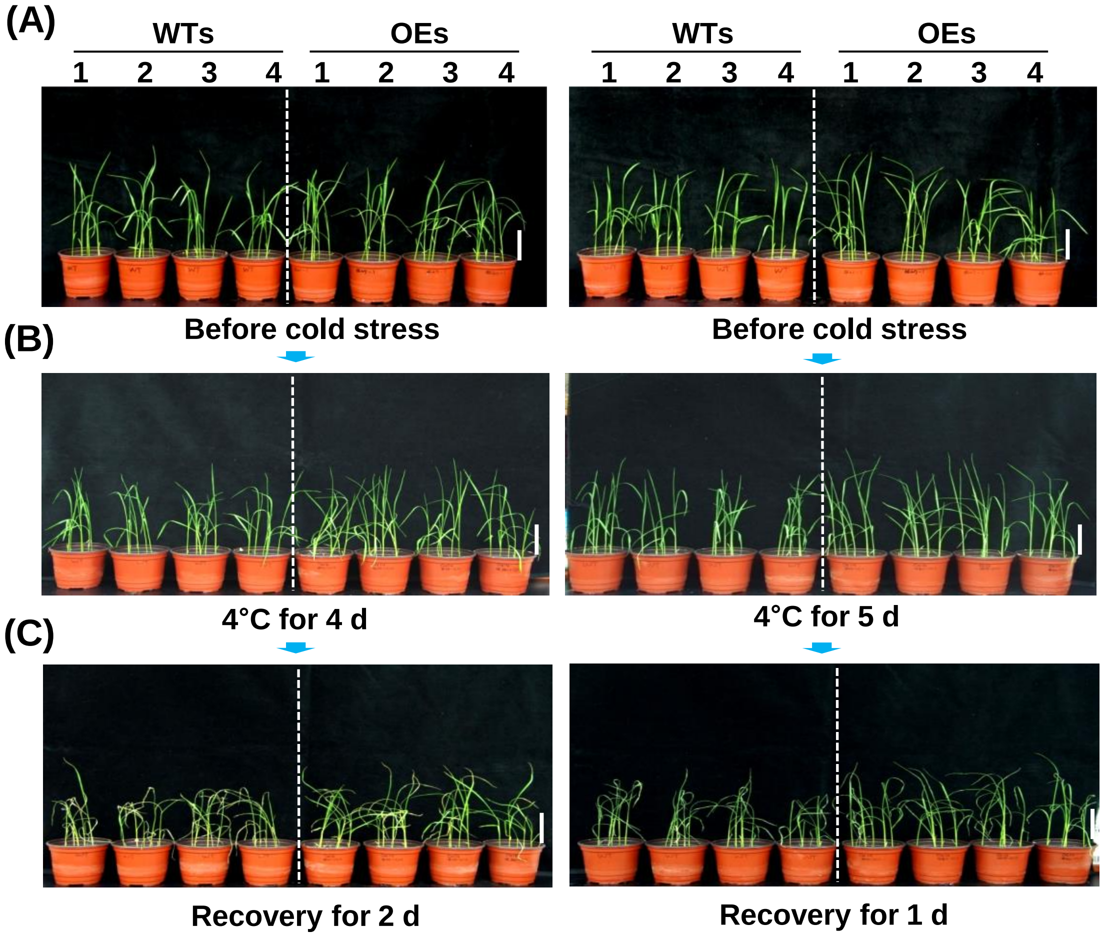

Figure S7. Response of *OsCYP19-4*-overexpressing rice plants to cold stress. (A) Two-week-old wild-type and OsCYP19-4 OE transgenic rice plants at the same stage before cold treatment. (B) Plants were subjected to 4°C for 4 or 5 d. (C) Following the cold treatment as in B, plants were allowed to recover in a growth chamber under normal growth conditions for 2 or 1 d, as indicated. WT1-4, different wild-type plants; OE1-4, independent OsCYP19-4 OE transgenic lines. Scale bars = 5 cm.

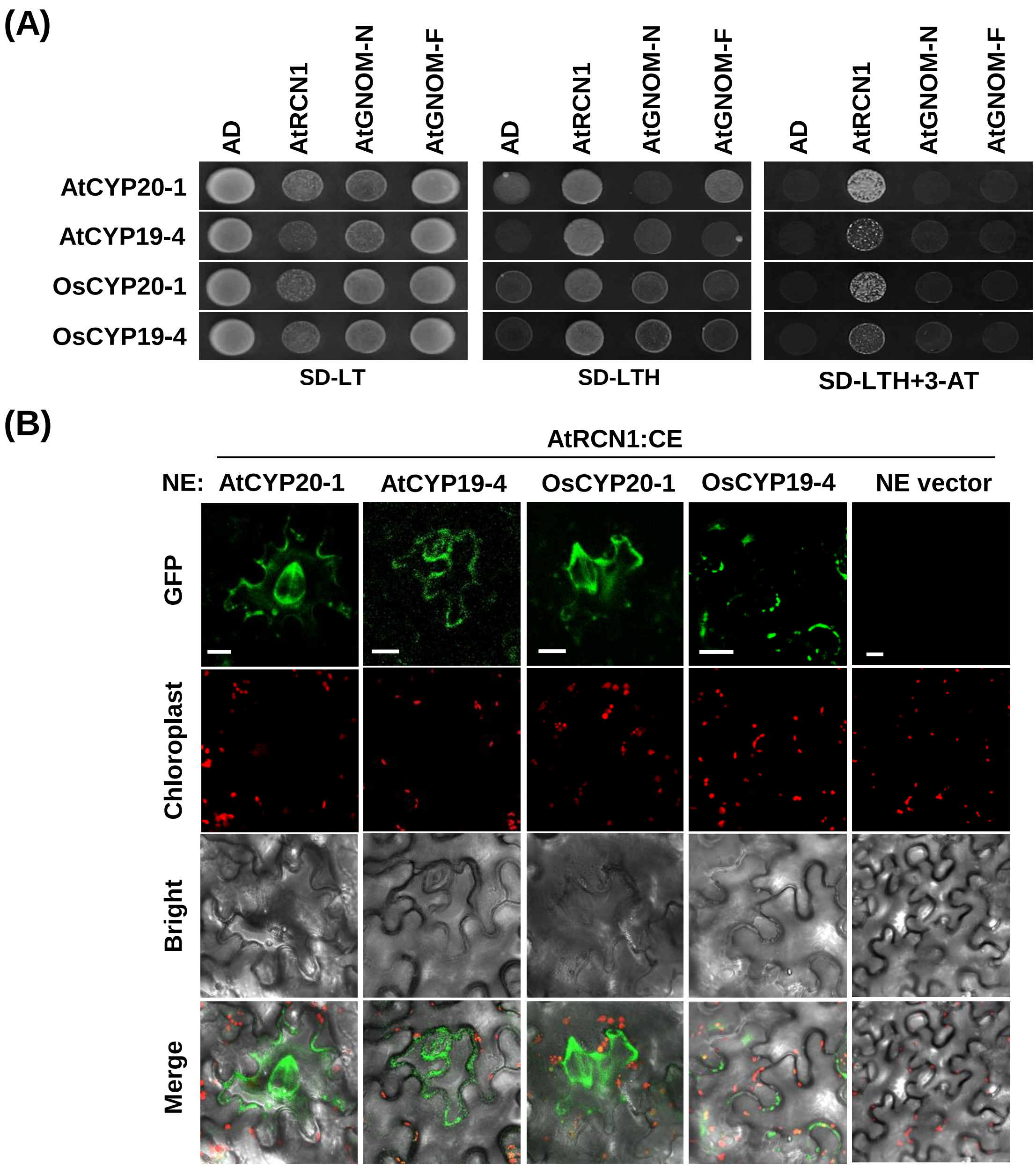

Figure S8. Interactions of CYP20-1s and CYP19-4s with AtRCN1. (A) Yeast two-hybrid analysis of CYP20-1s and CYP19-4s with AtRCN1, AtGNOM N-term (AtGNOM-N;1-250 aa) and AtGNOM full length (AtGNOM-F;1-1451 aa). Transformed yeast cells were grown onto synthetic dextrose (SD) media lacking leucine and tryptophan (-LT) or leucine, tryptophan and histidine (-LTH) and supplemented with 1.5 mM 3-AT. (B) Interaction of CYP20-1s and CYP19-4s with AtRCN1 using Bimolecular fluorescence complementation (BiFC). *Agrobacterium tumefaciens* GV3101 containing the BiFC constructs were co-infiltrated into *N. benthamiana* leaves. The leaves were observed for yellow fluorescent protein (YFP) fluorescence at 24–48 h post-injection. NE vector was used as a negative control. Scale bars = 20 μm.

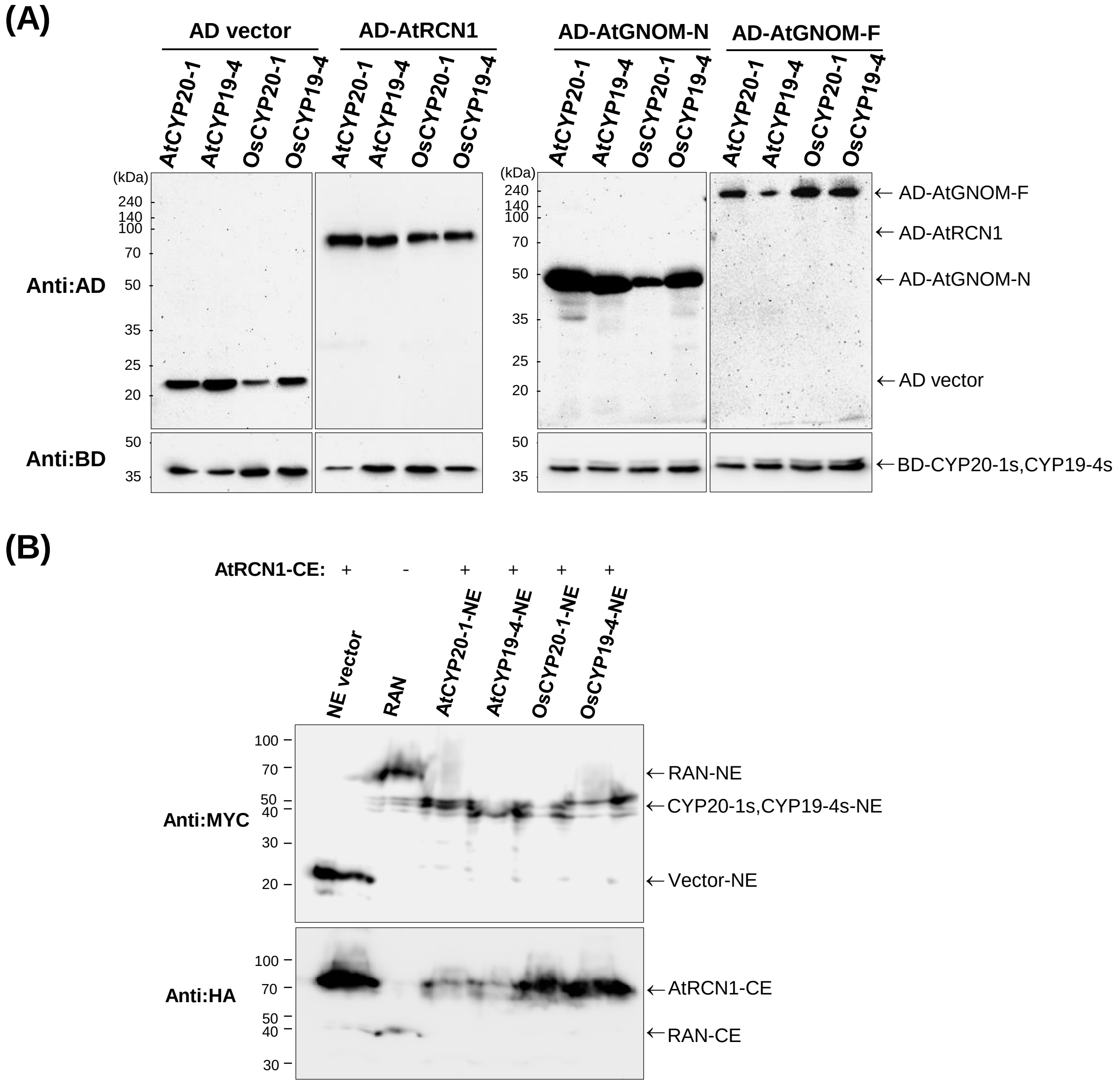

Figure S9. Immunoblot assay of Y2H and BiFC constructs. (A) Proteins were extracted from each yeast cells grown on liquid SD-LT media. Gal4-activation domain (AD) fused AtRCN1, AtGNOM-N, AtGNOM-F proteins were detected with anti-AD antibody. Gal4-binding domain (BD) fused CYP20-1s and CYP19-4s proteins were detected with anti-BD. (B) Proteins were extracted from *N. benthamiana* leaves after 3 days agroinfilteration. YFP N-term (NE) fused RAN, CYP20-1s and CYP19-4s were detected with anti-MYC antibody. YFP C-term (CE) fused AtRCN1 and RAN were detected using anti-HA antibody.
